# A pennycress *transparent testa 8* knockout mutant has drastic changes in seed coat anatomy and chemical compositions

**DOI:** 10.1093/jxb/erag078

**Published:** 2026-02-13

**Authors:** Xinxin Ding, Summer Duckworth, Madeline Southworth, Andrew Lipton, Chaevien S Clendinen, Barsanti Gautam, Maliheh Esfahanian, Dusan Velickovic, John C Sedbrook, Pubudu Handakumbura

**Affiliations:** Environmental Molecular Sciences Laboratory (EMSL), Pacific Northwest National Laboratory (PNNL), Richland, WA 99354, USA; Environmental Molecular Sciences Laboratory (EMSL), Pacific Northwest National Laboratory (PNNL), Richland, WA 99354, USA; Environmental Molecular Sciences Laboratory (EMSL), Pacific Northwest National Laboratory (PNNL), Richland, WA 99354, USA; Environmental Molecular Sciences Laboratory (EMSL), Pacific Northwest National Laboratory (PNNL), Richland, WA 99354, USA; Environmental Molecular Sciences Laboratory (EMSL), Pacific Northwest National Laboratory (PNNL), Richland, WA 99354, USA; School of Biological Sciences, Illinois State University, Normal, IL, USA; School of Biological Sciences, Illinois State University, Normal, IL, USA; Environmental Molecular Sciences Laboratory (EMSL), Pacific Northwest National Laboratory (PNNL), Richland, WA 99354, USA; School of Biological Sciences, Illinois State University, Normal, IL, USA; Environmental Molecular Sciences Laboratory (EMSL), Pacific Northwest National Laboratory (PNNL), Richland, WA 99354, USA; University of Szeged, Hungary

**Keywords:** Cell wall, metabolomics, pennycress (*Thlaspi arvense* L.), proanthocyanidins, seed aging, seed anatomy, seed coat, seed coat permeability, seed imbibition rates, solid-state NMR

## Abstract

Pennycress is a winter annual intermediate crop with ∼30% seed oil content suitable for producing biofuels. Here, we evaluated seed development, anatomy, and agronomically relevant traits of a *transparent testa 8* knockout mutant (*tt8-2bp*) generated by CRISPR genome editing to improve seed quality. We performed histochemical analyses on wild-type and *tt8-2bp* seeds at different developmental stages. No visible anatomical defects were observed in embryos and endosperm of *tt8-2bp* seeds. However, *tt8-2bp* seed coats had drastically reduced proanthocyanidins and proanthocyanidin monomers, which correlated with increased seed coat permeability, increased imbibition rates, and altered seed aging of *tt8-2bp* seeds. A cuticle layer was detected in *tt8-2bp* and wild-type seed coats. Further analysis is required to assess possible quantitative and structural defects in the *tt8-2bp* seed cuticle. Based on metabolomic and solid-state NMR analyses, *tt8-2bp* seed coats had decreased aromatic compounds and cell wall polysaccharides compared with wild-type seed coats. Consistently, *tt8-2bp* seeds also had reduced non-embryonic tissue dry weights, increased embryo dry weights, and unchanged total seed weights compared with wild-type seeds. This indicated altered nutrient partitioning during *tt8-2bp* seed development. The agronomic implications of *tt8-2bp* altered seed traits on pennycress domestication are discussed in depth.

## Teaser

Histochemical analyses of pennycress seeds revealed proanthocyanidin deficiency in *tt8-2bp* seed coats accompanied by increased seed coat permeability, increased imbibition rates, and altered seed aging compared with wild-type seeds.

## Introduction

Pennycress (*Thlaspi arvens*e L.) is an emerging diploid oilseed crop in the *Brassicaceae* family, notable for its high cold tolerance and short life cycle, which makes it suitable as a winter intermediate crop between corn and soybean rotations (Basnet and Ellison, 2024; Lee *et al*., 2024). Pennycress seeds are rich in oil (29–32% DW) and protein (33–46% DW) (Moser *et al*., 2009; Tsogtbaatar *et al*., 2015; Chopra *et al*., 2019; Arias *et al*., 2023), making them valuable for biofuel production from seed oil and animal feed production from seed meal (Moser *et al*., 2009; Phippen *et al*., 2022). A draft genome of pennycress has been assembled (Dorn *et al*., 2015), and comparative analyses revealed a high similarity of the protein-coding genes between pennycress and Arabidopsis, which helped with pennycress gene function annotations and studies (Chopra *et al*., 2018). Additionally, pennycress can be transformed via *Agrobacterium*-mediated vacuum infiltration, enabling genetic modification and genome editing (McGinn *et al*., 2019). The annotated genome and molecular tools have facilitated rapid domestication of pennycress through translational research.

Improving pennycress seed quality and oil content is central to its domestication (Chopra *et al*., 2020; Esfahanian *et al*., 2021; Jarvis *et al*., 2021; Hartnell *et al*., 2023; Guzha *et al*., 2024). Seed quality, particularly seed germination and vigor, directly influences seedling establishment, crop density, canopy development, and yield (Kumar *et al*., 2016; Reed *et al*., 2022). However, pennycress seeds often exhibit dormancy, especially when freshly harvested (Hazebroek and Metzger, 1990; Chen *et al*., 2022). While dormancy can be broken with gibberellic acid treatment or storage at room temperature for at least 1 month (Hazebroek and Metzger, 1990; Sedbrook *et al*., 2014; Koirala *et al*., 2022), these methods increase processing costs and delay planting, making reduced dormancy a key trait for pennycress domestication. Additionally, increasing seed oil content is crucial for the economic value of pennycress. For example, a 1% reduction in seed oil content in soybeans can result in a US$20 million revenue loss in the USA (Digrado *et al*., 2024). Studies in *Brassica napus* (e.g. rapeseed and canola) showed only weak correlations between seed germination and oil contents (Gu *et al*., 2019; Brown *et al*., 2023, 2024), indicating that it is feasible to improve both traits simultaneously.

Knockout (KO) mutations in several Arabidopsis *TRANSPARENT TESTA* (*TT*) genes, including *TTG1* (Chen *et al*., 2015), *TT2* (Chen *et al*., 2012), and *TT8* (Chen *et al*., 2014), lead to increased seed oil content and reduced dormancy (Debeaujon *et al*., 2000). In Arabidopsis, *TTG1*, *TT2*, and *TT8* encode transcription factors which form a transcription factor complex and synergistically regulate the flavonoid biosynthesis pathway (Baudry *et al*., 2004). This TTG1–TT2–TT8 transcription factor complex directly activates the *BANYULS* gene, which encodes a core enzyme for proanthocyanidin (PA) biosynthesis in the seed coat inner integument and the chalaza, where PAs and PA monomers accumulate (Debeaujon *et al*., 2003). PA monomers are flavanols, and the most common ones found in plant seeds are catechin and epicatechin (Ayadi *et al*., 2022; Yu *et al*., 2022). PA and PA monomers are colorless flavonoid polymers which turn brown upon oxidation, and higher degrees of PA polymerization are accompanied by increased hydrophobicity (Xie *et al*., 2003; Feng *et al*., 2014).

KO mutations in pennycress *TTG1*, *TT2*, and *TT8* genes also produce transparent seed coats, indicating their conserved roles in PA biosynthesis (Chopra *et al*., 2018). Pennycress *tt2* and *tt8* KO mutants both displayed reduced dormancy similar to Arabidopsis *tt2* and *tt8* KO mutants (Debeaujon *et al*., 2000; Ott *et al*., 2021). Notably, pennycress *tt8* KO seeds also exhibit higher oil and protein contents and a lower fiber content compared with the wild type (Sedbrook and Durrett, 2020; Koirala *et al*., 2023; Gautam *et al*., 2026). Additionally, phylogenetic, gene expression, and functional analyses indicated that *TT8* and some other *TT* genes play pivotal roles in crop seed evolution and domestication, affecting seed coat properties, dormancy, and post-zygotic reproductive barriers (Zumajo-Cardona *et al*., 2023a, b; Qian *et al*., 2025). In Arabidopsis, the post-zygotic reproductive barrier inhibits plants generating viable seeds from interploidy crosses, but *tt8* KO mutants fully overcome this reproductive barrier (Zumajo-Cardona *et al*., 2023a). If this function of *TT8* is conserved in other crops with triploid block, *tt8* KO mutants can allow for interploidy crossing and facilitate transfer of desirable traits including improved seed nutrient profiles, disease resistance, and stress tolerance. In summary, these results identify *TT2* and *TT8* as promising targets for enhancing pennycress seed quality and the nutrient profile. However, to effectively utilize these mutations for domestication, comprehensive studies on their impacts on seed development, anatomy, and key agronomic traits are needed. Such research is essential not only for evaluating effects of *tt* KO mutants but also for advancing our understanding of the cellular and molecular mechanisms modulating seed traits in pennycress, laying the groundwork for further improvement of seed quality.

Seed development and anatomy of Arabidopsis have been well characterized and served as the reference for this study. In angiosperms, the embryo and endosperm derive from a double fertilization event while the seed coat arises from the integuments of the ovule (Lafon-Placette and Köhler, 2014; Huang *et al*., 2023). After fertilization, the embryo, endosperm, and seed coat each follow distinct developmental programs. Embryo development occurs in two phases: morphogenesis, during which the apical–basal pattern of the basic embryo body is established; and maturation, during which the embryo accumulates storage compounds and undergoes dehydration (Jürgens, 2001; Huang *et al*., 2023). The endosperm development also has two main phases, namely the syncytial phase and the cellular phase, where the endosperm initially undergoes mitosis without cell wall formation and the cellularization occurs later (Lafon-Placette and Köhler, 2014; Huang *et al*., 2023). As the embryo matures, most endosperm cells undergo programmed cell death to provide space and nutrients for embryo growth, leaving only the peripheral endosperm layer alive at seed maturity (Huang *et al*., 2023). In contrast, the seed coat forms exclusively from maternal tissues. Most angiosperms have two integument layers, the inner and outer integuments, that surround the embryo sac. In Arabidopsis, the inner integument (ii) consists of three layers (ii1, ii1′, and ii2) and the outer integument (oi) has two (oi1 and oi2), which both eventually undergo programmed cell death (Huang *et al*., 2023). The ii1 layer accumulates PAs which impart the brown color of wild-type seeds, enhance seed coat mechanical strength, and regulate seed coat permeability.

Here, we analyzed a pennycress *tt8* KO mutant (*tt8-2bp*), generated *via* CRISPR/Cas9 [clustered regularly interspaced palindromic repeats (CRISPR)/CRISPR-associated protein 9] editing in the Spring32-10 (Spring32) background, an inbred line notable for minimal dormancy and vernalization independence, and thus frequently used for lab research (Gautam *et al*., 2026). The *tt8-2bp* mutant was selected from >20 *tt8* KO mutants showing similar phenotypic changes (Gautam *et al*., 2026). The *tt8-2bp* mutation introduced a 2 bp deletion in the *TT8* gene, causing pronounced alterations in seed coat anatomy and chemical compositions, as revealed by histological staining. The most notable change of *tt8-2bp* seed coats was a significant reduction in PAs and PA monomers, correlating with increased seed coat permeability, faster imbibition rates, and altered seed aging compared with the wild type. To investigate how changes in *tt8-2bp* seed coat chemical compositions may be linked to the altered seed traits, we compared metabolite profiles of non-embryonic tissues (NETs; including seed coats and tightly associated endosperm) of *tt8-2bp* and the wild type using LC-MS. The flavonoid and phenylpropanoid pathways were severely disrupted in *tt8-2bp*, with significant decreases in PAs, PA monomers, and lignin monomer precursors. Spatial metabolomics and solid-state NMR (ssNMR) confirmed the drastic reduction of PAs and PA monomers, and revealed decreased cell wall polysaccharides in *tt8-2bp* seed coats. Consistent with these findings, mature *tt8-2bp* seeds exhibited lower NET dry weights but higher embryo dry weights, indicating that *tt8* KO mutations altered nutrient partitioning between NETs and embryos in pennycress. This study highlights the central role of *TT8* in regulating pennycress seed coat anatomy and metabolism which in turn affects seed traits and nutrient partitioning between NETs and embryos. We discussed in depth the implications of our study on seed trait engineering for improved seed germination, aging, and nutrient profiles in pennycress and related oilseed crops.

## Materials and methods

### Plant growth

The *tt8-2bp* KO allele contained a 2 bp deletion located 179 bp downstream of the start codon and caused a frameshift mutation (Gautam *et al*., 2026). Mature seeds of Spring32-10 wild-type (Spring32) and *tt8-2bp* (*tt8*) were first germinated in Petri dishes before transfer into pots. Pennycress seeds were sterilized by rinsing three times with 70% ethanol followed by three rinses with sterile Milli-Q water. For each rinse, 3 s of vortex were applied. Then, sterilized seeds were placed on autoclaved Whatman papers in sterile Petri dishes with 2 ml of sterile Milli-Q water added. Petri dishes were sealed with parafilm and placed in the same growth chamber which was later used to grow pennycress plants for 2–4 d. Germinated seedlings were transferred to pots (8.9 cm W×8.9 cm L×8.9 cm D) containing pre-wetted PRO-MIX® BX Mycorrhizae™ medium. Pots were kept in trays (28 cm W×55 cm L×6.2 cm D, 18 pots in a full tray) which were covered with plastic domes for 2 d to maintain moisture. Plants were grown under a 16 h/8 h light/dark cycle at 22 °C/20 °C and 50%/70% humidity (day/night) with 200 μmol m^−2^ s^−1^ light intensity during the day. Watering and fertilization were done by adding deionized (DI) water and 100 ppm N liquid fertilizer (0.67 g l^–1^, Jack’s Professional #77220) to the trays. Before the third pair of true leaves emerged, each tray received 1 liter of DI water on Tuesdays and Fridays. Afterward, 1 liter of fertilizer was added on Fridays and, after bolting, on both Tuesdays and Fridays in addition to the 1 liter of DI water added to each tray. Once seed pods formed, 2 liters of DI water and 1 liter of fertilizer were added to each tray on Tuesday and Friday each week until seed harvest or until all the seed pods were senesced.

### Manual pollination and chemical fixation of pennycress seeds

To obtain seeds at various developmental stages, flowers were manually pollinated to ensure the exact timing of pollination, and seeds were collected on specific days post-pollination for histology and MALDI-MSI [matrix-assisted laser desorption ionization MS imaging] analyses. For seed histology, seeds were harvested at 7, 11, 15, 19, 23, and 27 days after pollination (DAP) and, for MALDI-MSI, seeds were harvested on 27 DAP. Based on earlier studies on pennycress flowers and seeds (Tsogtbaatar *et al*., 2015; Thomas *et al*., 2017), flowers are larger than those of Arabidopsis but have very similar structures, and thus can be pollinated manually in the same way as done with Arabidopsis flowers. To ensure seed sample consistency, only flowers from the lead stem were pollinated at 6–10 d after the opening of the first flower, and each flower was only pollinated once. All the seed samples were harvested between 14.00 h and 17.00 h to ensure sample consistency. For histological analysis, seed pods were harvested at defined stages, and seeds were fixed in a formalin:ethanol:acetic acid:water solution (10:50:5:35%, v/v/v/v) with 4% formaldehyde. After seeds were submerged in the fixation solution in glass vials, 15 min of vacuum infiltration were applied, and the glass vials were sealed and stored at 4 °C until analysis. For MALDI-MSI analysis, seed pods were harvested at 27 DAP and seeds were fixed by flash-freezing in liquid nitrogen. The samples were stored at −80 °C until analysis.

### Paraffin embedding of fixed pennycress seeds and histological analyses

Chemically fixed seeds were transferred into 60% ethanol in a Petri dish. Five small punctures were made on each side of the seeds to allow paraffin infiltration, as intact seed coats blocked paraffin entry. Seeds were then dehydrated through a graded ethanol series, cleared in xylenes, and embedded in paraffin wax (Supplementary Table S1). Sections (8 μm thickenss) were cut with a Mcrom Heidelberg rotary microtome (Model HM330) and mounted on microscope glass slides labeled with pencil so that the labels would not fade during deparaffinization and staining. Slides were incubated at 57 °C for 1 h to allow paraffin sections to adhere tightly to glass slides.

Before staining, sections were deparaffinized in xylene and rehydrated through a decreasing alcohol series (Supplementary Table S2), in which the final alcohol concentration matched that of the staining solution. For unstained controls, sections were rehydrated with DI water, then dehydrated in 100% ethanol and xylenes, and mounted with Permount (SP15-100, Fisher Chemical) before being covered with coverslips. All samples were examined using a ×10, ×20, or ×40 objective (Zeiss Fluar ×10/0.50, Zeiss LD Plan-Neofluar ×20/0.4 Corr M27, and Zeiss LD Plan-Neofluar ×40/0.60 Corr). Brightfield and fluorescence images were captured with a Zeiss Axiocam 305 color camera and a Zeiss AxioCam Cc1 fluorescence camera, respectively, on a Zeiss PALM Microbeam Laser Capture Microdissection System. Staining conditions for different dyes are described below.

Seed paraffin sections were stained with alcian blue and safranin O to visualize primary and secondary cell walls. After rehydration to 50% ethanol, sections were stained for 1 min with 1.5% safranin O in 50% ethanol, rinsed four times in DI water (20 s each), then stained with 1% alcian blue (3% acetic acid, pH 2.5) for 3 min, and followed by a 1 min DI water rinse. Finally, sections were differentiated in 95% ethanol for 1.5 min, dehydrated in 100% ethanol and xylenes (1 min each), and mounted with Permount before being covered with coverslips.

For visualization of primary cell wall components and starch granules, sections were stained with alcian blue and Lugol’s iodine (Kutík and Beneš, 1977). After rehydration to 50% ethanol and a 3 min DI water incubation, sections were stained with 1% alcian blue, rinsed, and differentiated as above. After incubation in DI water for 1 min, sections were stained with 0.25% Lugol’s iodine (VIG-271, Volu-Sol) for 7 min and rinsed twice (20 s each) in DI water. Sections were mounted with a customized medium (40% Arabic gum, 0.05% Lugol’s iodine, 59.95% DI water, w/w/v) to iodine-stained starch, which can be stored at 4 °C in the dark for up to 1 week. Stained sections were visualized immediately or after overnight drying in the dark.

Seed paraffin sections were stained with phloroglucinol-HCl (i.e. Wiesner staining solution; 2% phloroglucinol, 12% HCl, 21% water, 65% ethanol, w/v/v/v) to visualize lignin (Pradhan Mitra and Loqué, 2014). The normality of HCl in the Wiesner staining solution was 3 N. Seed sections were first rehydrated to 70% ethanol. After applying 1–2 drops of the staining solution, sections were covered with a coverslip and imaged within 10 min before the staining solution dried up.

For PA detection, sections were deparaffinized with xylenes, incubated in 90% methanol for 5 min, then stained with freshly prepared vanillin-HCl solution (1% vanillin, 90% methanol, 3.7% HCl, 5.3% DI water, w/v/v/v) (Feng *et al*., 2014; Xuan *et al*., 2014). The normality of HCl in the vanillin-HCl solution was 1.2 N. Sections were covered with a coverslip and visualized within ∼10 min before the staining solution dried up.

To detect pectin in cell walls, paraffin sections were first deparaffinized with xylenes, rehydrated to 50% ethanol, and incubated in DI water for 5 min. Then, seed sections were stained with 0.05% (w/v) ruthenium red (MilliporeSigma, 11103-72-3) in DI water, rinsed twice in DI water (20 s each), dehydrated in 100% ethanol and xylenes (1 min each), and mounted with Permount before being covered with a coverslip.

For cuticle detection, seed sections were stained with either 0.06% Sudan IV (w/v in 60% ethanol) or 0.01% auramine O (w/v in 0.05 M Tris–HCl, pH 7.2). For Sudan IV staining, sections were deparaffinized with xylenes, rehydrated to 70% ethanol, stained for 10 min in 0.06% Sudan IV, rinsed in 50% ethanol and DI water (10 s each), and mounted with 50% glycerol (v/v in 1× phosphate-buffered saline, pH 7.4). For auramine O staining, sections were deparaffinized with xylenes, rehydrated to 50% ethanol, incubated in DI water for 5 min, stained for 3 min in 0.01% auramine O, rinsed twice in DI water (1 min each), and mounted with 50% glycerol. Auramine O fluorescence was visualized using a cyan fluorescent protein (CFP) filter set (Zeiss Filter Set 47 HE).

### Cryosectioning and staining of 15 DAP and mature pennycress seeds to detect proanthocyanidins

Pennycress wild-type and *tt8-2bp* seeds at 15 DAP were flash-frozen immediately after harvest and stored at −80 °C until analysis. Before analysis, mature seeds were imbibed in Milli-Q water for 24 h at 4 °C in darkness, punctured twice (once on each side of the seeds), and vacuum infiltrated with Milli-Q water for 15 min. Mature and 15 DAP seeds were then embedded in a pre-chilled embedding medium [7.5% hydroxypropyl methylcellulose (HPMC), 2.5% polyvinylpyrrolidone (PVP), 90% Milli-Q water, w/w/v] kept on ice. A thin layer of embedding medium was poured into a mold and chilled on dry ice; seeds were placed on this layer before it fully froze. Then, the seeds were covered with more embedding medium and kept on dry ice until frozen completely. Embedded samples were stored at −80 °C until sectioning. The embedded seeds were sectioned at −12 °C using the CryoStar NX-70 Cryostat (Thermo Scientific, Runcorn, UK), and 25 μm sections were thaw mounted on glass microscope slides. The slides were vacuum dried in a desiccator immediately after sectioning. To visualize PAs, two drops of freshly made vanillin-HCl solution (1% vanillin, 90% methanol, 3.7% HCl, 5.3% DI water, w/v/v/v) was added on top of the sections. For unstained control sections, two drops of HCl solution (90% methanol, 3.7% HCl, 5.3% DI water, v/v/v) were added on top of the sections.

### Mature seed mucilage staining

Mature pennycress wild-type and *tt8-2bp* seeds were stained with ruthenium red following a published protocol for staining Arabidopsis seed mucilage (McFarlane *et al*., 2014), using Col-0 Arabidopsis seeds as positive controls. On the day of staining, 0.01% ruthenium red (Sigma-Aldrich, 11103-72-3) and 50 mM EDTA (pH 7.5) solutions were prepared. Seeds were placed in 2 ml Eppendorf tubes with 800 μl of 50 mM EDTA and shaken at 400 rpm for 2 h at room temperature to release mucilage. After removing the EDTA solution, 800 μl of 0.01% ruthenium red solution was added and incubated for 1 h at room temperature with shaking at 400 rpm. The staining solution was then replaced with Milli-Q water, and seeds were immediately examined under a dissecting microscope.

### Seed coat permeability measurement

Mature seeds were harvested from seven randomly selected plants from each genotype, wild type and *tt8-2bp* (i.e. seven biological replicates). From each plant, three sets of 50 seeds were imbibed in 1 ml of Milli-Q water, 20 mg ml^−1^ safranin O in Milli-Q water, or 20 mg ml^−1^ toluidine blue O in Milli-Q water, respectively, at 4 °C for 4 d. After the dissolved dyes were removed and seeds were thoroughly rinsed with Milli-Q water, 10 seeds per biological replicate were randomly sampled, dissected, and observed under a microscope for counting stained embryos.

### Seed imbibition rate measurement

Mature seeds of the wild type and *tt8-2bp* were harvested from seven plants per genotype (i.e. seven biological replicates per genotype). From each biological replicate, ∼200 mg of seeds were weighed and placed in 2 ml Eppendorf tubes with 1 ml of Milli-Q water. Tubes were vortexed for 1 min, centrifuged at 2000 *g* for 3 min to submerge the seeds, and then incubated at 4 °C for a total of 24 h. Seeds were weighed after 1, 2, 3, 4, 6, and 24 h of imbibition. After each time point, water was removed, and seeds were blotted dry, weighed, and then returned to the tubes. Water was added, and tubes were vortexed and centrifuged before continued imbibition at 4 °C. The imbibition rate (Si) after any duration of the imbibition period was calculated as Si=(Wi−Wb)/Wb, where Wi and Wb are seed weights after and before imbibition. Statistical analyses were done to assess differences in imbibition rates between the wild type and *tt8-2bp* at each time point.

### Seed accelerated aging test

Seed accelerated aging tests are commonly used to evaluate seed quality. Usually, seed germination rates are tested after aging seeds at 35–45 °C and 76–100% relative humidity for 24 h to several weeks, depending on the species and testing purpose (Kibinza *et al*., 2006; Rajjou *et al*., 2008; Coelho *et al*., 2022; Silva *et al*., 2025). Mature wild-type and *tt8-2bp* seeds from six plants per genotype (i.e. six biological replicates per genotype) were grown and harvested at the same time. About 30 seeds per biological replicate were randomly sampled and subjected to accelerated aging for 48 h at 38 °C and 90% humidity in darkness. For each genotype and treatment (with or without accelerated aging), 25 seeds per biological replicate were randomly sampled. All seeds were surface sterilized by rinsing seeds twice with 70% ethanol and then twice more in sterile Milli-Q water. Surface-sterilized seeds were air-dried in a biosafety cabinet for 1 h before being imbibed overnight in sterile Milli-Q water at 4 °C. Afterwards, seeds were planted (25 seeds per 7.6×7.6 cm pot) in pre-wetted PRO-MIX® BX Mycorrhizae™ medium on top of a piece of sterilized filter paper 1 cm from the top of the pots. After planting, pots were filled completely with growth medium, saturated with DI water, placed in trays with 500 ml of DI water at the bottom, and covered with clear plastic domes. The day after planting was counted as day 1, and cotyledon emergence was recorded every other day up to 12 d after planting.

### Metabolite extraction and liquid chromatography–mass spectrometry

Developing (27 DAP) and mature (>35 DAP) wild-type and *tt8-2bp* pennycress seeds were used for metabolite extraction. We followed established best practices for metabolomic sample handling (Dong *et al*., 2016). Briefly, the developing seeds were collected, snap-frozen, and stored in sealed tubes in darkness at −80 °C, and the mature seeds were stored in sealed Eppendorf tubes at 4 °C until analysis. Samples were processed ∼1 year after harvest. For the LC-MS metabolomic analysis, we used five biological replicates per genotype and developmental stage. NETs were manually isolated from the developing and mature seeds under a dissecting microscope. All dissected tissues were carefully examined and rinsed three times with 1 ml of 70% ethanol followed by three times with 1 ml of ultrapure water to prevent sample cross-contamination.

Metabolites were extracted from the NETs. The metabolite extraction protocol was adapted from Handakumbura *et al*. (2019), Dewhirst *et al*. (2021), and Balasubramanian *et al*. (2024a) with some modifications. Isolated NETs were lyophilized for 24 h and ∼20 mg of dried tissues were used for downstream processing. Tissues with known weights were transferred into 2 ml safelock tubes, chilled in liquid nitrogen, ground using a Geno/Grinder (Cole-Parmer®), and extracted with 80:20 methanol:water (20 μl mg^−1^ dry tissue). Samples were then incubated at 21 °C (1200 rpm, 1 h) in an Eppendorf Thermomixer C (NO: 5382). Finally, the supernatant was saved after centrifugation at 12 000 *g* for 10 min, filtered through 0.2 μm PTFE membranes (Pall AcroPrep 96 Filter Plates), and analyzed via LC-MS.

Filtered samples were analyzed by LC-electrospray ionization tandem MS (LC-ESI-MS/MS) with a Thermo Vanquish Flex UHPLC system coupled to a Thermo QExactive HF Orbitrapmass spectrometer. All metabolite samples were separated using both reverse-phase C18 (RP C18 LC-MS) and hydrophilic interaction (HILIC LC-MS) chromatography to enable separation of hydrophobic, polar, and hydrophilic molecules. For RP C18 LC-MS, separation was performed on a Thermo Hypersil GOLD column (2.1×150 mm, 3 μm particle size) at 40 °C. The mobile phase A (water with 0.1% formic acid) and B [acetonitrile (ACN) with 0.1% formic acid] gradient for metabolite separation is given in Supplementary Table S3A. For HILIC LC-MS, separation was performed on an ACQUITY UPLC BEH HILIC column (2.1×100 mm, 1.7 μm particle size) at 50 °C (Clendinen *et al*., 2019). The mobile phase A (5% ACN, 95% 10 mM NH_4_OAc in H_2_O with 0.05% NH_4_OH) and B (100% ACN with 0.05% NH_4_OH) gradient for metabolite separation is provided in Supplementary Table S3B. After separation, samples were analyzed in both positive and negative ion modes using higher-energy collision dissociation. The heated ESI source parameters are set as follows: spray voltage 3.7 kV or 3.0 kV for positive and negative modes, respectively; capillary temperature 350 °C; S lens RF level 50 arbitrary units, and aux gas heater temperature 150 °C. Full MS scan data are acquired at a resolving power of 120 000 full width at half-maximum (FWHM) at *m/z* 200 with a scanning range of *m/z* 80–800. The automatic gain control (AGC) target is set at 3E6 ions, with a maximum injection time of 20 ms. The data-dependent acquisition (dd-MS2) parameters used to obtain product ion spectra are as follows: resolving power 15 000 FWHM at *m/z* 200, AGC target 1E5 ions with maximum injection time of 100 ms, isolation width 0.4 *m/z*, loop count 12, and normalized collision energy (NCE): 20, 30, and 40 eV.

### LC-MS data analysis

LC-MS raw data were processed using Thermo Compound Discoverer 3.3, with metabolite identification based on internal reference libraries and external databases (MzCloud, GNPS, MoNet, etc.). For RP and HILIC positive and negative modes, spectra were aligned using adaptive curves with maximum retention time shifts of 0.3 min or 0.6 min, respectively, and a 3 ppm mass tolerance. Peaks were selected with intensity ≥15 000 and chromatographic signal-to-noise ratios ≥3, then grouped based on a 3 ppm mass and 0.3 min retention time tolerance. Features were filtered depending on the number of samples. Compounds were assigned using isotopic patterns, retention time, MS1, and/or MS2. All identifications and peak integrations were manually validated before export for statistical analysis. Identification confidence was classified into four levels, of which level 1 represents the highest confidence: level 1 (matching MS1, MS2, and retention time), level 2 (matching with MS2 only), level 3 (matching with MS1 and retention time), and level 4 (matching primarily with MS1 with partial MS2 match). In this study, only molecules identified at confidence levels 1–3 were considered as differentially expressed (DE) metabolites after statistical analyses. Processed LC-MS data were exported to Excel worksheets with metabolite classification based on Kyoto Encyclopedia of Genes and Genomes (KEGG) database annotations and relative metabolite abundances in NETs detected under positive or negative modes (Supplementary Tables S4–S7).

The exported data were processed with an R package pmartR (version 2.4.5) (Stratton *et al*., 2019; Degnan *et al*., 2023) for data normalization and statistical tests. Principal component analysis (PCA) was done to check the quality of LC-MS data to ensure all biological replicates of the same sample type cluster together in a PCA plot (see the Results). Data were log2-transformed and median-normalized to reduce systematic biases introduced during sample processing. To compare the relative metabolite abundances in developing and mature NETs of *tt8-2bp* versus the wild type, independence of missing data ANOVA tests were applied with *P*-values adjusted for the false discovery rate (FDR) (Webb-Robertson *et al*., 2010; Stratton *et al*., 2019; Degnan *et al*., 2023). Metabolites were classified as DE with an FDR≤0.1 and the absolute value of log2-transformed fold change (|log2FC|) ≥0.58 (FC ≥1.5 for up-regulation and ≤0.67 for down-regulation in *tt8-2bp* NETs), following thresholds used in other metabolomic studies (Ho *et al*., 2012; Zimmermann *et al*., 2017). DE metabolites were identified for each LC-MS dataset (Supplementary Tables S8–S11) and combined into a complete list (Supplementary Table S12). DE metabolites showing opposite change patterns (up-regulated in one dataset, down-regulated in another) were excluded and considered as having no significant changes, while those consistently up- or down-regulated across datasets were listed once and their average log2FC values are listed in Supplementary Table S12.

### Seed MALDI-MSI analysis

Pennycress wild-type and *tt8-2bp* seeds were harvested at 27 DAP, flash-frozen, and stored at −80 °C until analysis. Three seed sections obtained from three different seeds of the wild type and *tt8-2bp*, respectively, were used for MALDI-MSI. Seeds were embedded as described earlier in ‘Cryosectioning and staining of 15 DAP and mature pennycress seeds to detect proanthocyanidins’. Cryosectioning, sample preparation, and MALDI-MSI protocols were adapted from Balasubramanian *et al*. (2024b) with minor modifications. All samples were sectioned at 20 μm thickness and thaw-mounted on indium tin oxide (ITO)-coated slides, with separate slides for positive and negative ion mode analysis. Sections were vacuum dried and coated with either 2,5-DHB (2,5-dihydroxybenzoic acid) or NEDC (*N*-naphthylethylenediamine dihydrochloride) as MALDI matrices using an M5-Sprayer for positive and negative ionization modes, respectively. DHB (40 mg ml^–1^ in 70% MeOH) was sprayed at 50 µl min^−1^ with a 70 °C nozzle temperature for 12 cycles with 3 mm track spacing and a criss-cross pattern. A 2 s drying period was added between cycles. NEDC (7 mg ml^–1^ in 70% MeOH) was sprayed at 120 µl min^−1^ with a 70 °C nozzle temperature for eight cycles with 3 mm track spacing and a criss-cross pattern. No drying was needed between cycles. For both DHB and NEDC matrices, the linear flow was set to 1200 mm min^−1^ with 10-PSI nitrogen gas, and the spray was applied at 40 mm nozzle height. Imaging was performed on a Bruker Daltonics 12T solariX FTICR mass spectrometer with a ParaCell. This instrument has an Apollo II ESI and MALDI source with a SmartBeam II frequency-tripled (355 nm) Nd:YAG laser (Bremen, Germany). MALDI-MSI data under positive and negative ionization modes were acquired from *m/z* 100 to 500 for small molecules with a 110 k mass resolution (*m/z* 400). Ion images were generated with FlexImaging (Bruker Daltonics, v.5.0) with a 25 μm step size (i.e. a 25×25 μm^2^ spatial resolution). The ion images were annotated automatically based on the centroided dataset using the METASPACE platform, a spatial metabolomics knowledge database (Ovchinnikova *et al*., 2020). For this study, the KEGG-v1 database was used to annotate the small molecule within 3 ppm *m/z* tolerance.

MALDI-MSI used in this study did not measure absolute metabolite abundances, only relative metabolite abundances. Relative metabolite abundance comparisons between wild-type and *tt8-2bp* seeds were based on MALDI-MSI from three biological replicates (i.e. sections from three different seeds per genotype). Whole seed and embryo regions were manually selected in METASPACE using brightfield images, and ion intensities of small molecules were exported. Total ion intensities and total areas of the tissue(s) where ion signals localized (i.e. pixel counts) were calculated for whole seed and embryo regions, with NET areas determined by subtracting embryo areas from whole seed areas. To compare the relative abundances of a specific molecular feature in wild-type versus *tt8-2bp* seed sections, the average ion intensity per pixel was calculated for NETs in each seed section. The average ion intensities were subsequently used for statistical analyses. There were no significant differences between the total pixel numbers of any seed compartment when comparing wild-type versus *tt8-2bp* seed sections.

### Solid-state NMR analysis of the mature seed outer seed coats

Mature pennycress wild-type and *tt8-2bp* seeds were collected from three plants per genotype, with 30 seeds randomly chosen from each plant (90 seeds per genotype) for ssNMR analysis. Seeds were rinsed three times with 70% ethanol, followed by three rinses with Milli-Q water, then soaked in 1 ml of Milli-Q water at 4 °C for 24 h to soften seed coats. Under a dissecting microscope, outer seed coats (i.e. mainly cell walls of seed coat outer integuments) were isolated manually in Milli-Q water, cut into ∼1 mm^2^ pieces, transferred to 2 ml Eppendorf tubes, and stored at −80 °C until ssNMR analysis. For each genotype, 57 mg of fully hydrated outer seed coats was used. SsNMR spectra were acquired from two biological replicates per genotype, consisting of ∼57 mg of hydrated outer seed coats pooled from mature wild-type and *tt8-2bp* seeds harvested from different plants as described above. Each spectrum was averaged over 65 536 scans and the two biological replicates of the wild type and *tt8-2bp* showed consistent results. One set of representative spectra are shown in the Results, consistent with common practice in the field (Marassi *et al*., 1997; Wang *et al*., 2016; Zhao *et al*., 2021).

SsNMR was performed at 9.4 T (399.86 MHz for ^1^H, 100.55 MHz for ^13^C) on an Agilent VNMRS spectrometer (Environmental Molecular Sciences Laboratory, Pacific Northwest National Laboratory) using a custom 4 mm MAS probe tuned to ^1^H/^13^C. The MAS housing (Revolution NMR) was fabricated from Kel-F for minimal ^13^C background signal. The rotors are standard zirconia sleeves with double o-ring Kel-F spacers (Revolution NMR). Carbon chemical shifts were referenced to a secondary standard of the methylene peak of adamantane at 38.48 ppm relative to tetramethylsilane at 0 ppm. Signal generation of ^13^C was through cross-polarization (CP) (Pines *et al*., 1972) with a standard ramped CP pulse sequence using a 3.0 µs ^1^H 90° pulse, a 1 ms contact pulse with a ramped ^1^H RF amplitude, and a 2 s recycle delay (Metz *et al*., 1994). SPINAL-64 decoupling was applied at an 83 kHz ^1^H nutation frequency (Fung *et al*., 2000). The lack of a background signal was verified using pure KBr under identical experiment conditions to those for the seed coat samples.

### Dry weight measurements of mature embryos and seed coats

Mature wild-type and *tt8-2bp* pennycress seeds were harvested from six plants per genotype (i.e. six biological replicates each), all grown and collected at the same time. Seeds were stored in sealed 1.5 ml Eppendorf tubes for ∼1 year before analysis. For each plant, two sets of 75 seeds were randomly selected: one set for whole seed weight measurements and the other for embryo and non-embryonic tissue weight measurements, respectively. NETs included seed coats and endosperm. Whole seed weights were measured after 24 h lyophilization. To measure NET dry weights, seeds were cleaned, imbibed, and dissected in DI water. Isolated embryos and NETs were then lyophilized for 24 h and weighed separately. Statistical analyses were done to compare dry weights and embryo-to-NET ratios of wild-type versus *tt8-2bp* seeds.

### Statistical analyses

All Student’s *t*-tests and one-way ANOVA tests were done with R (version 4.3.3) (R Core Team, 2024). The significance threshold was *P*-value ≤0.05 if not otherwise specified.

For comparing areas of *tt8-2bp* versus wild-type embryos and whole seeds, seed and embryo areas were measured from 8–9 seeds per genotype (one paraffin section per seed per DAP) at 7, 11, 15, 19, 23, and 27 DAP, respectively. Sections were prepared and imaged as described in ‘Paraffin embedding of fixed pennycress seeds and histological analyses’. Areas (μm^2^) were quantified manually using Fiji’s Measure tool (Schindelin *et al*., 2012). Two-tailed Student’s *t*-tests were done to compare the areas of *tt8-2bp* versus wild-type embryos and whole seeds, respectively, on each DAP.

For comparing seed imbibition rates, seven biological replicates were used for the wild type and *tt8-2bp*, respectively. Two-tailed Student’s *t*-tests were done to compare the seed imbibition rates of wild-type versus *tt8-2bp* seeds after each period of imbibition.

To test how seed accelerated aging treatment affected wild-type versus *tt8-2bp* seeds, we performed two-tailed Student’s *t*-tests comparing their cotyledon emergence rates on different days after planting for seeds with and without 48 h aging treatment, respectively.

For comparing dry weights of whole seeds, embryos, and seed coats/endosperm as well as seed water contents, two-tailed Student’s *t*-tests were used for measurements collected from six biological replicates of wild-type and *tt8-2bp* seeds, respectively.

For MALDI-MSI analysis, average ion intensities of three biological replicates (i.e. MALDI-MSI data collected from seed sections of three different seeds) were used in comparisons of all the molecular features of interest between wild-type and *tt8-2bp* seed sections at 27 DAP. The log2-transformed average ion intensities were used in two-tailed Student’s *t*-test to determine if there was a statistically significant difference between wild-type and *tt8-2bp* seeds. The significance threshold was *P*-value ≤0.1.

For DE fatty acids and lipids identified by LC-MS, one-way ANOVA tests were performed on log2-transformed FCs of the major fatty acid species up- or down-regulated in *tt8-2bp* embryos and NETs, respectively, to determine if there are significant differences in FCs among different fatty acid species.

## Results

### No anatomical defects in endosperm and embryo development of *tt8-2bp* seeds

**Fig. 1.**
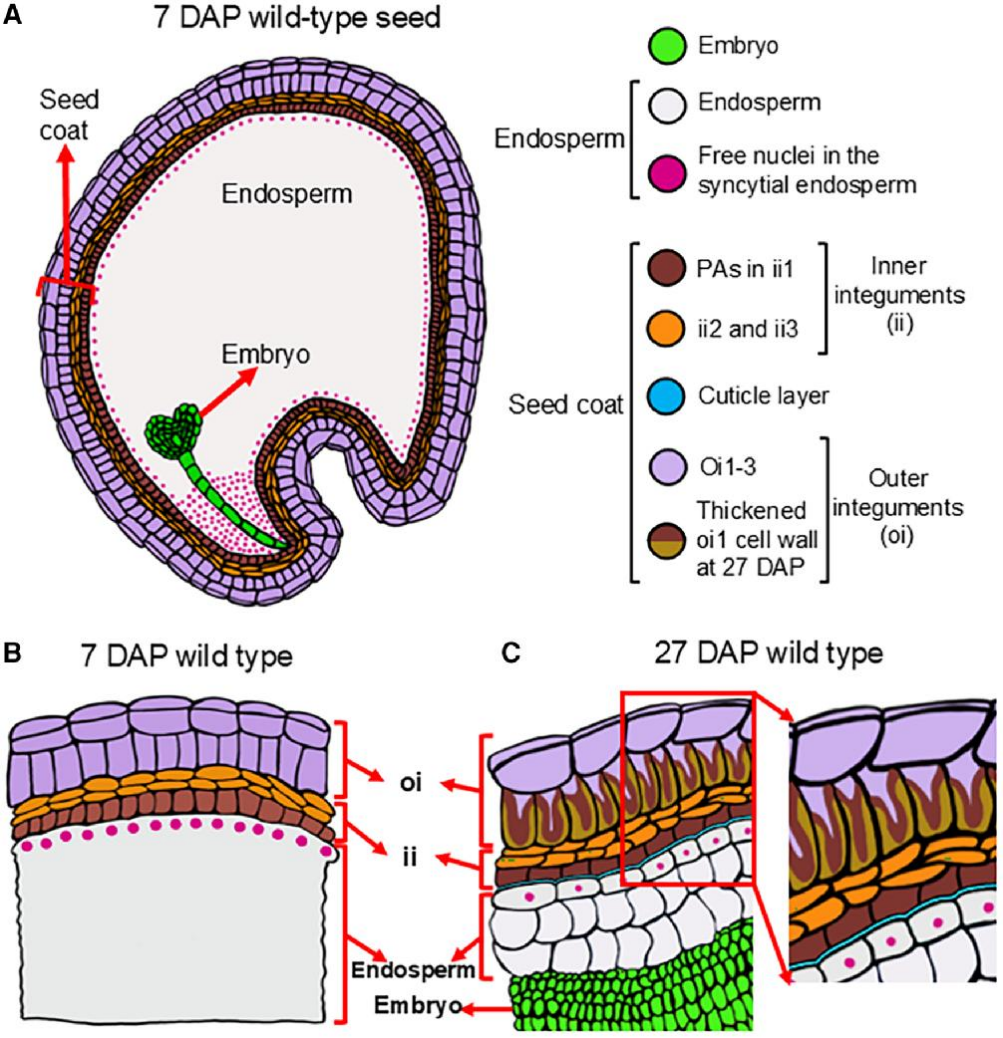
Diagrams depict the anatomy of Spring32 wild-type pennycress seeds, emphasizing seed coat structure at 7 and 27 DAP. (A) A diagram shows the anatomy of the cross-section of a 7 DAP pennycress seed. (B) A diagram shows the seed coat cell layers and endosperm at 27 DAP. Note that the ii1 layer adjacent to the endosperm accumulates PAs and PA monomers. (C) A diagram shows the seed coat, endosperm, and embryo at 27 DAP. Note the cuticle layer between the endosperm and the PA-accumulating ii1 layer, and the thickened oi1 cell wall containing PAs and PA monomers adjacent to ii cell layers. In (B) and (C), the ii and oi layers are numbered 1, 2, and 3 from the embryo side to the seed surface.

In this study, we characterized the anatomy of developing Spring32 wild-type and *tt8-2bp* KO mutant seeds from 7 to 27 DAP. To provide context, we first summarize the anatomy of the wild-type seed and seed coat. At 7 DAP, the wild-type embryo is at the heart stage and the endosperm is syncytial, with free nuclei visible (deep-pink dots in Fig. 1A, B). The seed coat consists of three ii layers and three oi layers, with ii1 accumulating PAs and PA monomers (Fig. 1B, C). At 7 DAP, ii and oi cells have thin primary cell walls (black lines in Fig. 1B). As development proceeds, the embryo enlarges rapidly and the endosperm undergoes cellularization. By 27 DAP, the embryo fills most of the seed, and oi1 cells develop thickened walls (Fig. 1C), eventually forming the hardened seed coat in mature seeds that protects the endosperm and embryo. Additionally, a cuticle layer is formed between the ii1 cell layer and the outermost endosperm cell layer by 27 DAP (the blue line in Fig. 1C).

Given the importance of *tt8* KO mutations in pennycress domestication (Gautam *et al*., 2026), it is essential to evaluate *tt8* seed traits, including anatomical changes, that may impact field performance. We compared the anatomy of *tt8-2bp* and wild-type seeds at 7, 11, 15, 19, 23, and 27 DAP when seed tissues undergo important transcriptional, metabolic, and morphological changes (Tsogtbaatar *et al*., 2015; Johnston *et al*., 2022). Flowers were manually pollinated, with 1 DAP marked as the day after pollination, and seeds were harvested and fixed for all the developmental stages to be studied (see the Materials and methods; Supplementary Fig. S1A). Under the growth condition of this study (see the Materials and methods), the siliques and seeds of *tt8-2bp* and the wild type grew rapidly until ∼19 DAP, when the siliques began to yellow and most leaves had fully senesced (Supplementary Figs S1B, S2A). By 27 DAP, the embryos of both genotypes and the seed coats of *tt8-2bp* turned a paler green, indicating reduced chlorophyll (Supplementary Fig. S2A). Meanwhile, wild-type seed coats became reddish-brown, suggesting PA oxidation.

**Fig. 2.**
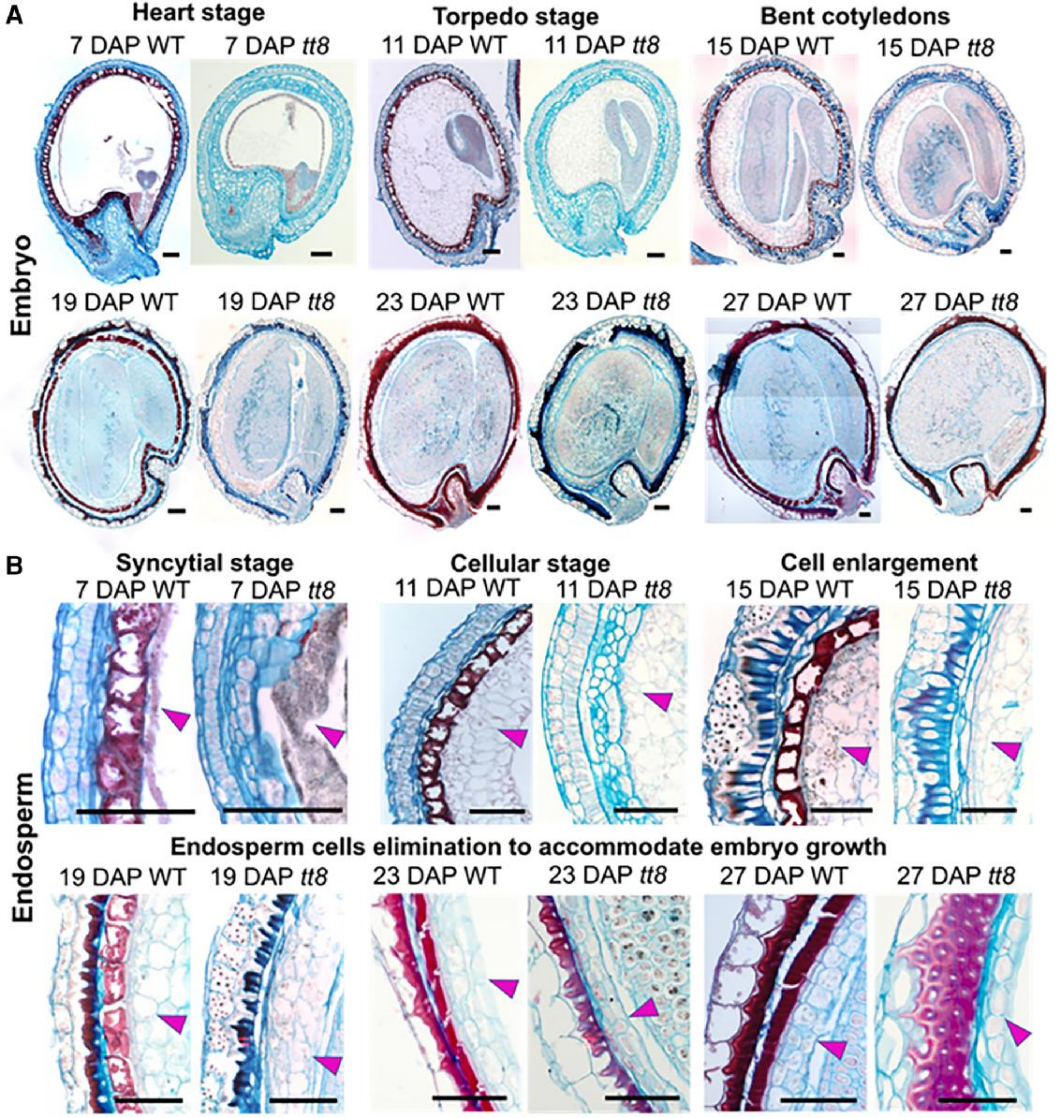
Histological staining of wild-type (WT) and *tt8-2bp* embryo and endosperm shows that the two seed tissues developed at a similar rate at 7–27 DAP. Developing seeds were fixed in 10% formalin, and 8 µm paraffin sections were obtained. Rehydrated sections were stained with safranin O (stains secondary cell wall and nuclei red) and counterstained with alcian blue (stains acidic polysaccharides of primary cell wall blue). The stained seed sections at 7–27 DAP show that the embryo (A) and endosperm (B) develop at a similar rate. The magenta arrowheads point to the endosperm cells. At least three seeds from three different plants were studied for each developmental stage. Scale bars=100 µm. The image of WT bent cotyledons 15 DAP is repeated in Supplementary Fig. S3A.

Histological staining was performed on *tt8-2bp* and wild-type seed sections to compare their anatomies at different developmental stages. For seed histological staining, fixed seeds were dehydrated, embedded in paraffin, and sectioned (see the Materials and methods). Sections were stained with safranin O and alcian blue, which have different affinities for various cell wall components (Blokhina *et al*., 2017; Santos *et al*., 2023). Alcian blue primarily stains acidic polysaccharides in primary walls, whereas safranin O stains proteoglycans and lignified secondary walls (Baldacci-Cresp *et al*., 2020; Pang *et al*., 2023). At least three seeds from three different plants were analyzed for each developmental stage. Stained seed sections at 7, 11, 15, 19, 23, and 27 DAP showed that embryo and endosperm development rates were similar in *tt8-2bp* and wild-type seeds, with no visible anatomical defects in *tt8-2bp*. The main seed tissues in a stained pennycress seed section are labeled in Supplementary Fig. S3. Both wild-type and *tt8-2bp* embryos were at the heart and torpedo stages at 7 and 11 DAP, respectively (Fig. 2A). At 7 DAP, the endosperm of both genotypes was syncytial as the endosperm nuclei were not surrounded by cell wall (Fig. 2B). At 11 DAP, the endosperm underwent cellularization and cell walls were visible (Fig. 2B). By 15 DAP, wild-type and *tt8-2* embryos had grown significantly in size and the cotyledons were fully bent (Fig. 2A; Supplementary Figs S2A, S4). From 15 to 27 DAP, wild-type and *tt8-2* embryos continued to grow and eventually took up most of the seed volume (Fig. 2A; Supplementary Fig. S4). During this period, wild-type and *tt8-2bp* endosperm appeared to gradually decrease in volume and cell layers (Fig. 2), indicating endosperm cell degradation to accommodate embryo growth in a way similar to Arabidopsis endosperm (Huang *et al*., 2023).

To determine the effect of *tt8* KO mutations on seed development, we quantified embryo and seed areas from cross-section images of developing seeds at 7–27 DAP similar to those shown in Fig. 2A. While *tt8-2bp* and wild-type embryos and seeds were comparable in size at 7–23 DAP, both embryo and seed areas of *tt8-2bp* were significantly reduced at 27 DAP (Supplementary Fig. S2B, C; Supplementary Table S13). This suggests that *tt8-2bp* and wild-type embryos and seeds grew at a comparable rate at 7–23 DAP. The reduction in *tt8-2bp* embryo and seed areas at 27 DAP coincided with the onset of seed coat senescence visible in both genotypes (27 DAP, Supplementary Fig. S2A), suggesting changes in late maturation processes of *tt8-2bp* seeds.

### The *tt8-2bp* mutation led to proanthocyanin deficiency and starch accumulation in ii1 cells

**Fig. 3.**
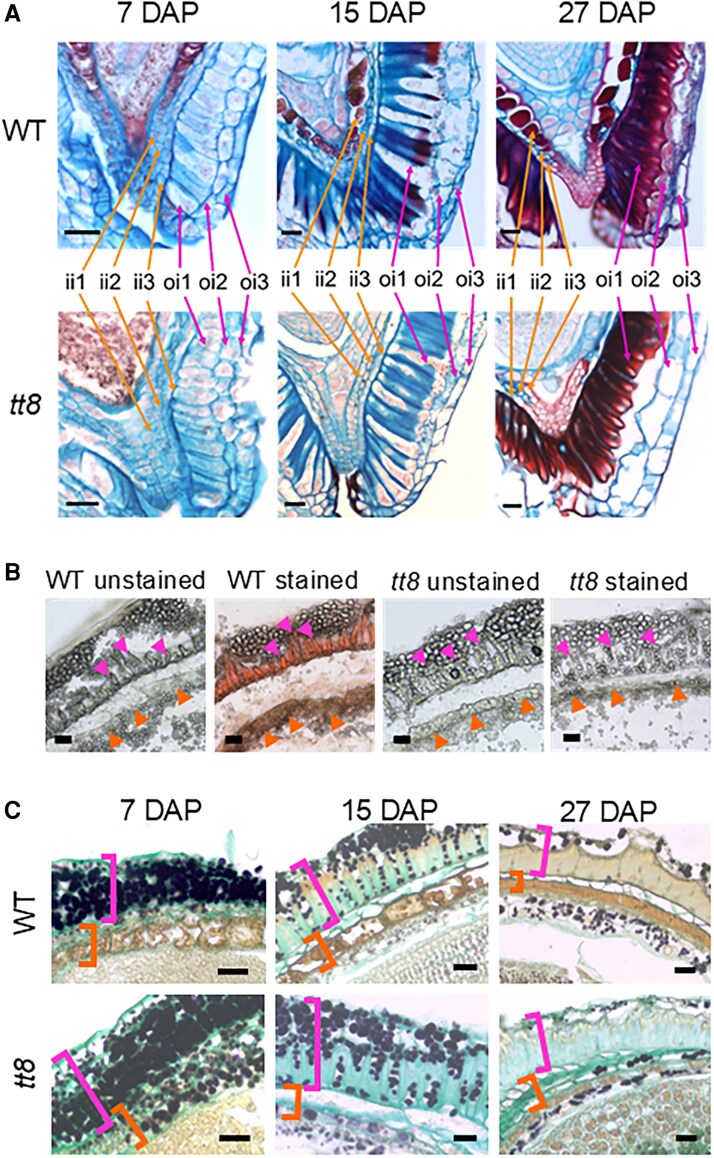
Three cell layers were found in the ii and oi of the Spring32 wild-type (WT) and *tt8-2bp* seed coats where the ii1 cells of the WT and *tt8-2bp* accumulate different metabolites during development. (A) Seed sections of the WT and *tt8-2bp* at 7, 15, and 27 DAP were stained with safranin O (stains secondary cell wall and nuclei red) and counterstained with alcian blue (stains acidic polysaccharides of primary cell wall blue). Note that only the WT ii1 cells stained red. (B) Seed cryosections of the WT and *tt8-2bp* at 15 DAP were stained with vanillin-HCl which stains PAs reddish-brown. The orange arrowheads point to the ii1 cells and the magenta arrowheads point to the thickened cell wall of oi1 cells. (C) Seed sections of the WT and *tt8-2bp* at 7, 15, and 27 DAP were stained with alcian blue and Lugol’s iodine which stain primary cell wall light turquoise and starch granules black, respectively. The orange brackets mark the ii1-3, and the magenta brackets mark the oi1-3. Note that the ii1 cells of *tt8-2bp* seeds accumulated starch granules. Scale bars=25 µm.

The Arabidopsis seed coat is composed of two distinct integuments: the ii with three cell layers and the oi with two cell layers (Haughn and Chaudhury, 2005). In Arabidopsis, the innermost ii layer (ii1) accumulates PAs while the outermost oi layer (oi2) accumulates mucilage (Windsor *et al*., 2000). Pennycress wild-type and *tt8-2bp* seeds both have three ii and oi layers (Fig. 3A), which we labeled 1–3 based on proximity to the embryo, consistent with Arabidopsis nomenclature. Although wild-type and *tt8-2bp* seeds have the same number of integument layers, seed coats of *tt8-2bp* lacked PAs and PA monomers in ii1 cells and exhibit changes in oi1 cell wall formation. In pennycress seeds, the ii and oi layers were distinguishable at the micropyle, where the three ii layers completely enclosed endosperm cells near the embryo root radicle whereas the oi layers left a small opening (Fig. 3A).

Pennycress wild-type ii1 cells accumulated PAs and/or PA monomers from 7 to 27 DAP, as indicated by some compounds stained dark red by alcian blue and safranin O, a feature absent in *tt8-2bp* ii1 cells (Fig. 3A). Since *TT8* is essential for PA biosynthesis in seed coat cells, the compounds stained dark red in pennycress wild-type ii1 cells are likely to be PAs and/or PA monomers (Fig. 3A). To detect PAs and PA monomers, we stained 15 DAP seed sections with the vanillin-HCl solution (see the Materials and methods). Vanillin-HCl staining is a simple and sensitive method for detecting PAs and PA monomers in plant tissues as they form red compounds upon reaction with vanillin (Deshpande *et al*., 1986; Feng *et al*., 2014; Xuan *et al*., 2014). When seed paraffin sections were stained, wild-type ii1 cells contained yellowish-brown metabolites in both unstained and vanillin-HCl-stained sections, but no colored metabolites were detected in *tt8-2bp* ii1 cells even after vanillin-HCl staining (Supplementary Fig. S5). While no red compounds were observed in vanillin-HCl-stained wild-type ii1 cells, we could not rule out that the yellowish-brown metabolites were PAs or PA monomers. PAs and PA monomers in the paraffin-embedded wild-type seeds could have been chemically modified during sample processing and thus did not react with vanillin-HCl. Therefore, we repeated the vanillin-HCl staining with wild-type and *tt8-2bp* seed cryosections where no chemicals were used for sample processing (see the Materials and methods). Red compounds were observed only in ii1 and oi1 of wild-type seeds (Fig. 3B), indicating accumulation of PAs and/or PA monomers in wild-type ii1 and oi1 only.

In contrast, the ii1 cells of *tt8-2bp* seeds accumulated uncolored granules (Fig. 3B). Since both wild-type and *tt8-2bp* seed coats appeared green during development (Supplementary Fig. S2A), the uncolored granules could be starch produced from photosynthates. Thus, wild-type and *tt8-2bp* seed paraffin sections were stained with alcian blue and Lugol’s iodine to visualize primary cell wall and starch granules (see the Materials and methods). After staining, the primary cell wall and starch granules were light turquoise and black, respectively (Fig. 3C). Starch granules were observed in oi cells of both genotypes, with a decreasing amount from 7 to 27 DAP. In ii cells of both genotypes, starch was present in ii2 and ii3 at 7 DAP but was undetectable at later stages. In *tt8-2bp* ii1 cells, starch granules were detected with a decreasing amount from 7 to 27 DAP (Fig. 3C), which showed that the *tt8* KO mutation caused PA deficiency and starch accumulation in pennycress seed coat ii1 cells.

### Distinct secondary cell wall compositions of oi1 cells in wild-type versus *tt8-2bp* seed coats

**Fig. 4.**
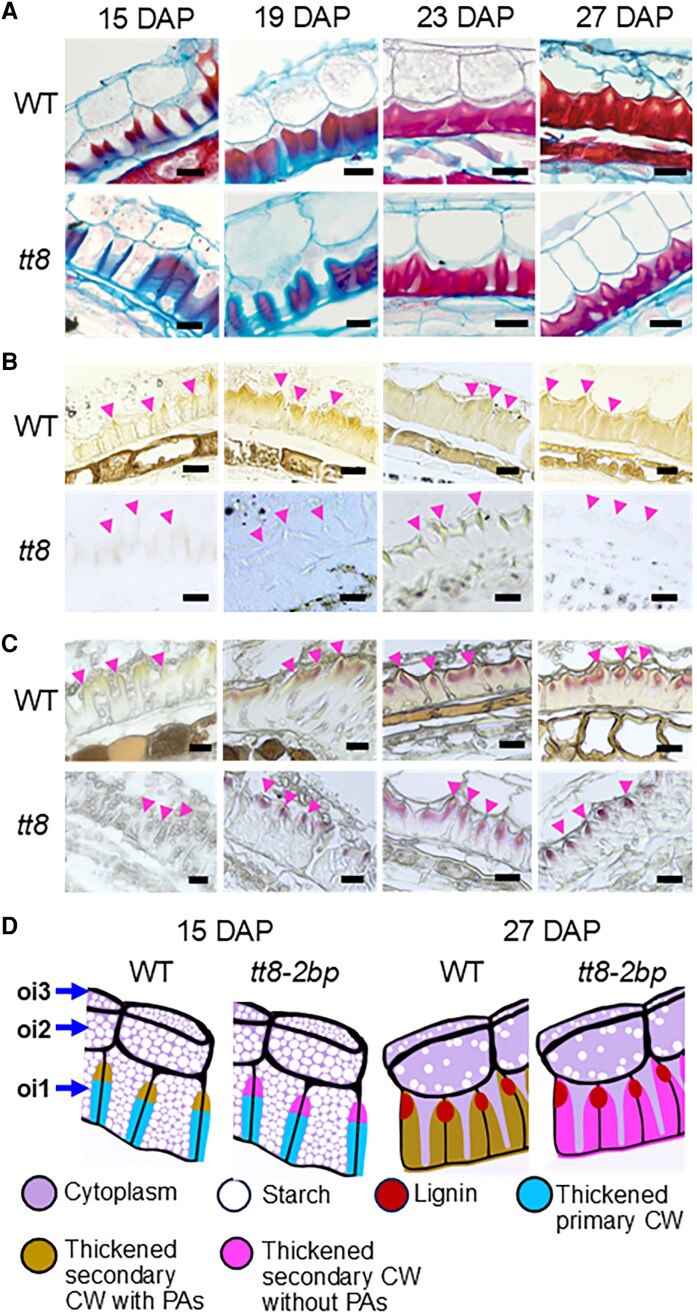
The thickened oi1 cell wall (CW) of Spring32 wild-type (WT) and *tt8-2bp* seeds have different chemical compositions at 15–27 DAP. (A) WT and *tt8-2bp* seed paraffin sections were stained with safranin O (stains the secondary CW and nuclei red) and counterstained with alcian blue (stains acidic polysaccharides of the primary CW blue). (B) WT and *tt8-2bp* seed paraffin sections were imaged without staining. Some brown pigments were observed in the thickened CW of the WT oi1 cells. (C) WT and *tt8-2bp* seed paraffin sections were stained with phloroglucinol-HCl to detect lignin which turned red after staining. (D) Diagrams of oi cell layers depicting anatomical changes in WT and *tt8-2bp* oi1 cells from 15 to 27 DAP. The black lines are the thin primary CW. In oi1 cells, the black lines indicate the locations of thin primary CW before CW thickening started. Magenta arrowheads in (B) and (C) point to the locations of the thickened oi1 CW closest to oi2 cells. Scale bars=25 µm.

In both wild-type and *tt8-2bp* seeds, oi1 cells began forming thickened cell walls between 11 and 15 DAP (Fig. 2B; Supplementary Fig. S4). From 15 to 27 DAP, cell wall thickening of oi1 was most pronounced in the anticlinal walls (perpendicular to the seed surface), while periclinal walls (parallel to the surface) adjacent to ii3 showed less thickening and those adjacent to oi2 cells showed no thickening (Fig. 4A, D). Oi2 and oi3 cell walls of wild-type and *tt8-2bp* seeds did not show any thickening at 15–27 DAP (Fig. 4A).

Wild-type and *tt8-2bp* seeds exhibited similar anatomical changes in oi layers from 15 to 27 DAP. The portion stained red in the thickened oi1 cell walls increased from 15 to 27 DAP, especially in the anticlinal cell walls (Fig. 4A). Because alcian blue stains the primary cell wall blue and safranin O stains the secondary cell wall red, this suggests that both wild-type and *tt8-2bp* seeds developed secondary cell wall during the oi1 cell wall thickening from 15 to 27 DAP. However, the chemical compositions of the thickened oi1 cell wall differed between the wild type and *tt8-2bp*. When stained with alcian blue and Lugol’s iodine, the *tt8-2bp* thickened oi1 cell wall turned a light turquoise color (Fig. 3C; Supplementary Fig. S6A), indicating the presence of acidic polysaccharides such as pectin in the secondary cell wall. This is not surprising since pectin is present in plant primary and secondary cell walls. In contrast, the thickened oi1 anticlinal walls of 27 DAP wild-type seeds did not stain by alcian blue but appeared yellowish-brown, similar to the color of the ii1 cells (Fig. 3C; Supplementary Fig. S6A). This is consistent with our earlier finding that PAs and/or PA monomers were accumulated in wild-type ii1 and oi1 cells (Fig. 3B), indicating the presence of PAs and/or PA monomers in the thickened oi1 cell wall of wild-type seeds.

We performed additional histochemical analyses to characterize the differences in the thickened oi1 cell wall between wild-type and *tt8-2bp* seeds. In unstained seed paraffin sections, the yellowish-brown metabolites (presumably PAs and/or PA monomers) were observed in ii1 and the thickened oi1 cell walls of wild-type seeds (Fig. 4B). In 15 DAP wild-type seeds, the yellowish-brown metabolites appeared to concentrate at the end of the oi1 anticlinal cell wall near oi2 (Fig. 4B, D). As the oi1 anticlinal cell wall gradually thickened, the yellowish-brown metabolites distributed more evenly throughout the oi1 anticlinal cell wall at 23 and 27 DAP (Fig. 4B, D). Because lignin is a critical component of secondary cell walls, we stained seed paraffin sections with phloroglucinol-HCl solution (see the Materials and methods), which stains lignin aromatic aldehydes pink or red (Pomar *et al*., 2002). Phloroglucinol-HCl staining showed that lignin deposition in the oi1 anticlinal wall began between 15 and 19 DAP in wild-type and *tt8-2bp* seeds, with no observable difference in lignin localization (Fig. 4C). In both genotypes, stained lignin aromatic aldehydes were detected in a small region of the thickened oi1 anticlinal cell wall near oi2 (Fig. 4C, D).

Arabidopsis seed coats accumulate mucilage consisting of complex pectinaceous polysaccharides (Western *et al*., 2000). The seed coat mucilage expands after absorbing water and extrudes to completely cover Arabidopsis seeds (Macquet *et al*., 2007a). Ruthenium red has high affinity for pectin, nucleic acids, and calcium-binding proteins (Steeling, 1970; Karpel *et al*., 1981; Cook *et al*., 2013), and is used routinely to detect seed mucilage. While an earlier study found that a natural pennycress variety lacks mucilage (Viudes *et al*., 2021), natural mutations can alter mucilage formation in Brassica seeds (Macquet *et al*., 2007b). Therefore, we tested mucilage release in Spring32 wild-type and *tt8-2bp* seeds. Wild-type and *tt8-2bp* pennycress seeds incubated in ruthenium red solution showed no extruded mucilage, whereas Arabidopsis seeds showed mucilage around mature seeds (Supplementary Fig. S6B). To verify the absence of mucilage in pennycress seed coat cells, wild-type and *tt8*-2bp seed paraffin sections were stained with ruthenium red. At 7, 15, and 27 DAP, ruthenium red stained the primary cell wall and oi1 secondary cell wall red in wild-type and *tt8-2bp* seeds (Supplementary Fig. S6C). No accumulation of water-soluble, pectin-rich mucilage in pennycress seed coat cells was observed (Supplementary Fig. S6C).

Based on the histochemical analyses, we summarized the developmental milestones and important anatomical features of oi cells in pennycress wild-type and *tt8-2bp* seeds (summarized in Fig. 4D). By 11 DAP, wild-type and *tt8-2bp* oi cells accumulated starch granules and had only primary walls. From 15 to 27 DAP in both genotypes, the amount of starch granules decreased, and oi1 cells developed thickened secondary walls, of which the anticlinal walls thickened more than the periclinal walls. In wild-type seeds, the thickened oi1 cell walls accumulated PAs and/or PA monomers which appeared to be absent in *tt8-2bp* seed coats. Lignin accumulation in the thickened oi1 cell wall began between 15 and 19 DAP and appeared to localize to small areas near oi2 in both genotypes. From 7 to 27 DAP, oi2 and oi3 cell walls did not thicken, suggesting that the thickened oi1 cell wall ultimately became the hardened protective coat of mature wild-type and *tt8-2bp* seeds.

### Increased seed coat permeability of *tt8-2bp* seeds linked to ii1 cell proanthocyanidin deficiency at micropyle and chalazal regions

**Fig. 5.**
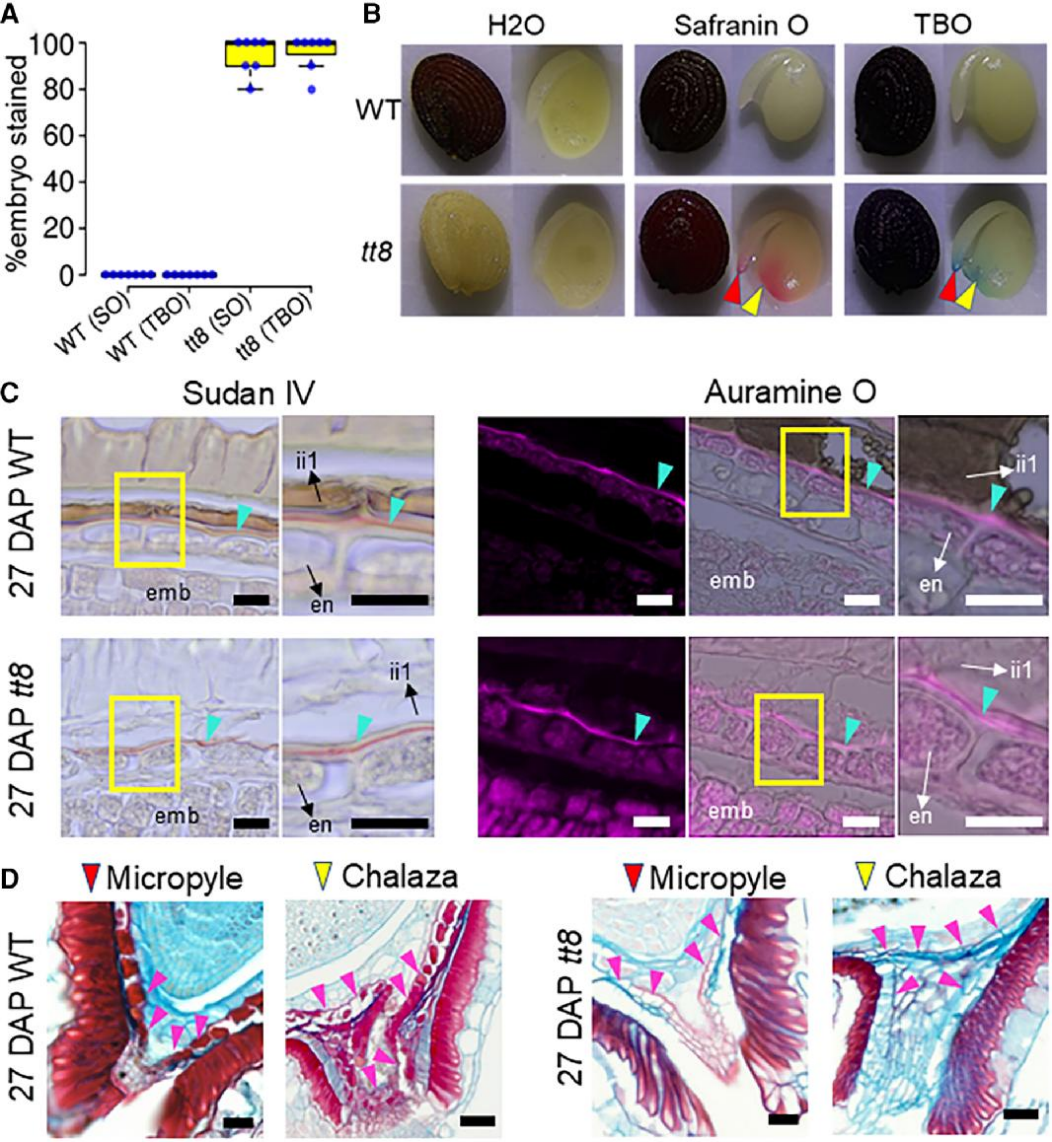
Assessing seed coat permeability of wild-type (WT) and *tt8-2bp* (*tt8*) mature seeds. (A) Percentages of embryos stained after mature WT and *tt8* seeds were submerged in water, safranin O (SO), or toluidine blue O (TBO) for 4 d. Quantification was done with seeds from six different plants of each genotype. (B) Representative images of WT and *tt8* seeds after submersion. *tt8* embryos were most heavily stained at the micropyle (red arrowhead) and chalaza (yellow arrowhead). (C) Sections of 27 DAP WT and *tt8* seeds stained by Sudan IV, a red dye, and auramine O, a fluorescent dye, to detect seed coat cuticle. Magnified images of the yellow box-labeled areas are shown next to the original images. Auramine O staining images from left to right are fluorescence images, overlay of fluorescence and brightfield images, and magnified images. Cyan arrowheads point to seed coat cuticle. En, endosperm; emb, embryo. Scale bars=20 µm. (D) Sections of 27 DAP WT and *tt8* seeds stained by alcian blue and safranin O show that the less permeable WT seed coat has PA-accumulating ii1 cells enclosing the micropyle and chalaza (magenta arrowheads). Scale bars=50 µm.

PAs are aromatic polymers whose hydrophobicity increases with their degree of polymerization (Xu *et al*., 2021). In *tt8-2bp* seed coats, the marked reduction of PAs and PA monomers can lead to decreased hydrophobicity, resulting in greater permeability to water-soluble molecules compared with wild-type seed coats. To evaluate this, we incubated seeds from seven different plants (i.e. seven biological replicates) per genotype in 20 mg ml^–1^ solutions of safranin O and toluidine blue O, respectively, at 4 °C for 4 d (see the Materials and methods). Significantly higher proportions of *tt8-2bp* seeds showed embryo staining with both dyes (Fig. 5A), especially near the micropyle and chalaza, indicating enhanced dye penetration through these seed coat regions (Fig. 5B). In contrast, mature wild-type pennycress seed coats accumulate oxidized PAs and/or PA monomers, imparting a reddish-brown color (Fig. 3B; Supplementary Fig. S2A). Whereas oxidized PA monomers are water soluble, oxidized PAs are more hydrophobic. Consistent with this, water did not turn brown following incubation or dissection of wild-type seeds, suggesting that hydrophobic PAs, rather than water-soluble PA monomers, predominate in wild-type seed coats.

To investigate the potential cause of the drastic increase in *tt8-2bp* seed coat permeability, we compared the seed coat cuticle and general anatomy of 27 DAP wild-type and *tt8-2bp* seeds at the micropyle and chalaza. To study seed coat cuticle, we stained seed paraffin sections with Sudan IV and auramine O (see the Materials and methods), respectively. Sudan IV, a red dye, and auramine O, a fluorescent dye, were used routinely to stain seed coat cuticle (De Giorgi *et al*., 2015; Loubery *et al*., 2018; Coen *et al*., 2019; Demonsais *et al*., 2020). Both dyes stained a thin layer of cuticle located between the ii1 layer of seed coats and the outermost layer of endosperm in the seed coats of 27 DAP wild-type and *tt8-2bp* seeds (Fig. 5C). This cuticle layer was also detected at the micropyle and chalaza of 27 DAP wild-type and *tt8-2bp* seeds, enclosing the endosperm and embryo (turquoise arrowheads in Supplementary Fig. S7). These results showed that *tt8-2bp* seeds were able to form the cuticle layer at the same location as the wild-type seeds. Additionally, we examined the seed anatomy of the micropyle and chalazal regions by staining 27 DAP seed paraffin sections with alcian blue and safranin O. The micropyle and chalaza of wild-type and *tt8-2bp* seeds were not completely enclosed within the thickened oi1 cell wall, exposing the ii layers to the outside environment (Fig. 5D). The PA-accumulating ii1 cells of wild-type seeds (magenta arrowheads in Fig. 5D) wrapped around the endosperm at the micropyle and chalaza, forming another barrier outside the seed coat cuticle layer. In contrast, most ii1 cells of *tt8-2bp* seeds (magenta arrowheads in Fig. 5D) only had primary cell wall, except for a few cells at the micropyle where their cell walls stained red, indicating secondary cell wall (Fig. 5D). It is likely that the thin-walled ii cells at the micropyle and chalazal regions of *tt8-2bp* seeds cannot effectively block water-soluble molecules from passing through their primary cell walls, contributing to the increased seed coat permeability.

### *tt8-2bp* seeds had increased imbibition rates and altered aging process

PA deficiency in *tt8-2bp* seed coats can also affect seed imbibition rates and the aging process due to alterations in seed coat hydrophobicity, permeability, and anti-oxidation properties. These traits can affect seed germination in the field and thus seed quality (Souza and Marcos-Filho, 2001; Chandra *et al*., 2020; Zhou *et al*., 2022; Xing *et al*., 2025).

**Fig. 6.**
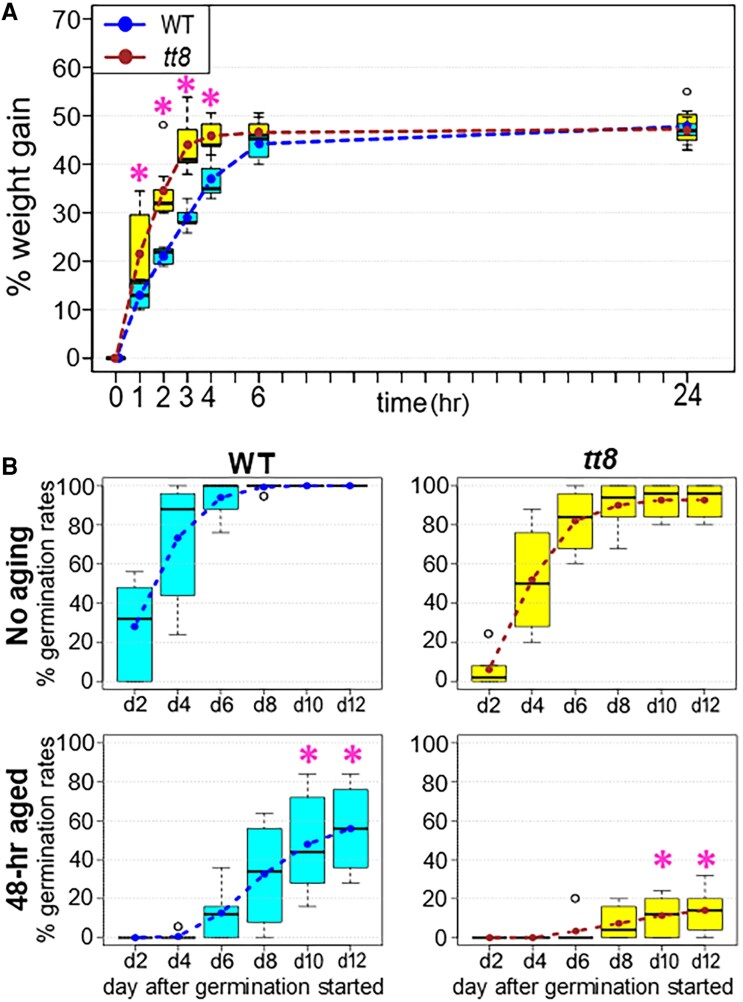
Boxplots of Spring32 wild-type (WT) and *tt8-2bp* (*tt8*) seed imbibition rates and germination rates of no aging and 48 h aged seeds. (A) Boxplots of seed imbibition rates after submerging seeds in water. Asterisks indicate a statistically significant difference (*P*-value ≤0.05) between the imbibition rates of the WT and *tt8* based on two-tailed Student’s *t*-tests. (B) Seed germination rates of seeds without aging and after 48 h aging treatment. Asterisks indicate a statistically significant difference (*P*-value ≤0.05) between the germination rates of the WT and *tt8* under the same aging condition on the same day after germination started based on two-tailed Student’s *t*-tests. Dotted lines (A) and (B) connect the mean values represented by boxplots.

To measure seed imbibition rates, we submerged seeds from seven different plants (i.e. seven biological replicates) of the wild type and *tt8-2bp*, respectively, for 1, 2, 3, 4, 6, and 24 h. The seed imbibition rates at each time point were calculated as percentages of absorbed water weights over seed weights before imbibition (see the Materials and methods). At 1, 2, 3, and 4 h after imbibition, *tt8-2bp* seeds had significantly higher imbibition rates than wild-type seeds (Fig. 6A). At 6 h and 24 h of imbibition, there was no significant difference in imbibition rates between wild-type and *tt8-2bp* seeds (Fig. 6A). This suggests that *tt8-2bp* seeds absorbed water faster at the early phase of imbibition but eventually absorbed the same amount of water as wild-type seeds when fully imbibed. After 24 h of imbibition, *tt8-2bp* and wild-type seeds on average absorbed water that was 47.3% and 47.9% of their weights before imbibition, respectively.

To assess seed aging, the accelerated seed aging test was used in which wild-type and *tt8-2bp* mature seeds were incubated at 38 °C, 90% humidity for 48 h. This test is widely used to evaluate seed tolerance to heat and high humidity during storage, and the results highly correlate with field emergence speed and final emergence rate of various crops (Matera *et al*., 2019; Favoretto *et al*., 2024). We used seeds harvested from six different wild-type and *tt8-2bp* plants (i.e. six biological replicates), respectively. We planted seeds without aging (no aging) and after 48 h aging treatment (48-h aged) at the same time and recorded cotyledon emergence every other day for 12 d after planting. We compared the emergence rates of the wild type and *tt8-2bp* on each day for seeds with and without aging treatment. There was no significant difference in emergence rates between the wild type and *tt8-2bp* under the no aging condition based on two-tailed Student’s *t*-tests (*P*<0.05, Fig. 6B). For seeds with aging treatment, *tt8-2bp* seeds had significantly lower emergence rates than wild-type seeds on day 10 and 12 based on two-tailed Student’s *t*-tests (*P*<0.05, Fig. 6B), indicating that *tt8-2bp* seeds aged faster under high heat and humidity.

### Metabolomic profiling revealed altered chemical compositions in endosperm and seed coats of *tt8-2bp* and the wild type

**Fig. 7.**
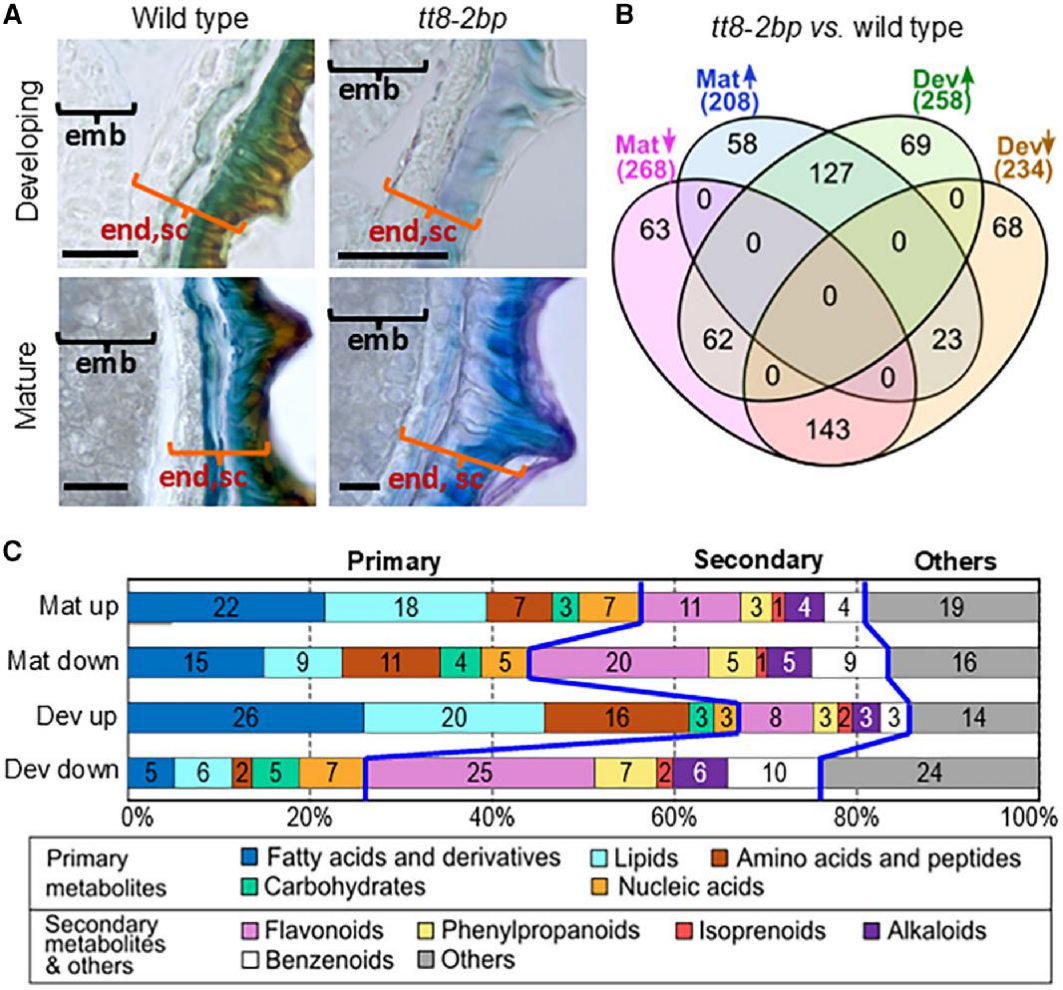
Overview of the LC-MS metabolomic results of the developing (dev) and mature (mat) non-embryonic tissues (NETs). (A) Toluidine blue O-stained developing (27 DAP) and mature (>35 DAP) seed cross-sections show the NETs used for LC-MS analysis [i.e. seed coats with attached endosperm (end, sc), and the embryo adjacent to the NETs]. Five biological replicates of NETs at the two different developmental stages were used for LC-MS for seeds of each genotype. Scale bars=50 µm. The wild-type mature image is repeated in Supplementary Fig. S10A. (B) Venn diagrams show overlaps of differentially expressed (DE) metabolites (*tt8-2bp* versus wild type) of mature (Mat) and developing (Dev) NETs. DE metabolites were identified by ANOVA tests with thresholds of FDR ≤0.1 and FC ≥1.5 or ≤0.67. (C) A bar chart shows percentages of up- and down-regulated DE metabolites (*tt8-2bp* versus wild type) in developing and mature NETs, respectively, belonging to different metabolite groups (i.e. primary metabolites, secondary metabolites, and others), separated by blue lines. The numbers in the bar chart are percentages of different DE metabolite groups. DE metabolites in the ‘others’ group include organic and inorganic acids, lignans, non-flavonoid polyketides, amines, acid esters, and ethers.

Histological analysis of 7–27 DAP seeds showed that *tt8-2bp* seed coats have altered anatomy and chemical compositions in the ii1 and oi1 layers. These changes resulted in increased seed coat permeability and seed imbibition rates, and accelerated aging under heat and humidity, potentially affecting seed longevity during storage and germination in the field. To investigate how altered seed coat chemical compositions affected these seed traits, we performed metabolomic profiling on NETs (composed of endosperm and seed coat) of *tt8-2bp* and wild-type seeds for quantitative comparison and metabolic pathway analysis. We selected seeds at 27 DAP (developing) to corroborate the histological data and seeds at >35 DAP (mature) to help compare seed coat compositions at maturity (Supplementary Figs S1, S2). NETs were analyzed collectively due to the tight association of endosperm and seed coat (specifically ii1) by the cuticle (Fig. 5C) which cannot be separated by manual dissection. For simplicity, we will refer to seeds and tissues at 27 DAP as ‘developing’ and at >35 DAP as ‘mature’ from this point on. The developing and mature NETs were mainly composed of 1–2 layers of endosperm cells, ii cell layers, thickened oi1 cell wall, and mostly empty oi cells (Figs 3A, 7A). Metabolites from NETs were extracted and analyzed via LC-MS.

Five biological replicates of NETs were collected from the developing and mature seeds of *tt8-2bp* and wild-type plants (see the Materials and methods). Metabolites were extracted and quantified by LC-MS using RP and HILIC separation methods under positive and negative ionization modes (Supplementary Tables S4–S7). PCA confirmed that all biological replicates clustered by genotype and developmental stage, demonstrating high consistency and reproducibility (Supplementary Fig. S8). DE metabolites in developing and mature NETs, respectively, were identified by comparing relative metabolite abundances in *tt8-2bp* versus wild-type NETs using ANOVA tests (FDR ≤0.1, FC ≥1.5 or ≤0.67; see Supplementary Tables S8–S12). Here, we describe the FC of a DE metabolite in *tt8-2bp* NETs as its abundance relative to wild-type NETs [e.g. metabolite-A has an increased abundance in *tt8-2bp* which was 2-fold of that in the wild type, meaning metabolite-A (*tt8-2bp*)=2×metabolite-A (wild type)]. Fewer DE metabolites were up-regulated in mature NETs than in developing NETs of *tt8-2bp* (208 versus 258), with 127 overlapping, while more DE metabolites were down-regulated in mature NETs compared with developing NETs of *tt8-2bp* (268 versus 234), with 143 overlapping (Fig. 7B). To identify metabolite groups and pathways most affected in *tt8-2bp* NETs, we summarized the numbers of DE metabolites belonging to primary metabolites, secondary metabolites, and some other metabolites not part of the former two groups (named ‘others’, Fig. 7C). Examples of DE metabolites in the ‘others’ group include organic and inorganic acids, lignans, non-flavonoid polyketides, amines, acid esters, and ethers (Supplementary Table S12). Primary and secondary metabolites together comprised the majority of DE metabolites in developing and mature NETs of *tt8-2bp*: 86% and 81% of up-regulated metabolites and 76% and 84% of down-regulated metabolites, respectively (Fig. 7C).

Fatty acids, lipids, amino acids, and peptides constituted the majority of DE primary metabolites in developing and mature NETs, both up- and down-regulated in *tt8-2bp* (Fig. 7C). Fatty acids and amino acids are the building blocks of lipids and proteins, respectively. Seed storage lipids and proteins are the most abundant storage compounds in oilseed embryos and endosperm (Baud and Lepiniec, 2010; Tsogtbaatar *et al*., 2015; Gacek *et al*., 2018). Most DE fatty acids and lipids in developing and mature NETs were fatty acids of various chain lengths and degrees of unsaturation and glycerophospholipids (Supplementary Table S12). Glycerophospholipids are the main components of the plasma membrane and membranes of organelles such as lipid bodies and protein storage vacuoles, among others. Some DE fatty acids and lipids are among the most abundant detected in wild-type seed oil, including erucic acid, linoleic acid, oleic acid, γ-linolenic acid (γ-linoleate), and a linoleoyl glycerol (storage lipid) with one linoleic acid chain (Jarvis *et al*., 2021). In developing *tt8-2bp* NETs, oleic acid, linoleic acid, and γ-linolenic acid were 5.6-, 2.3-, and 2.2-fold, respectively, of those in developing wild-type NETs. In mature *tt8-2bp* NETs, linoleic acid and γ-linolenic acid were 0.5- and 0.6-fold, respectively, of those in mature wild-type NETs, while linoleoyl glycerol was 2.3-fold of that in mature wild-type NETs. Erucic acid increased in *tt8-2bp* NETs at both developmental stages (2.3- and 2.4-fold of those in wild-type NETs). These DE fatty acids and lipids known to be abundant in seed oil were probably localized to the endosperm, the sole nutrient storage tissue in NETs. For DE amino acids and peptides, their protein origins and localizations within NETs cannot be determined by the LC-MS data alone.

Flavonoids and phenylpropanoids constituted most of the up- and down-regulated secondary metabolites in both developing and mature *tt8-2bp* NETs (Fig. 7C). This aligns with histological evidence indicating reduced PA accumulation in *tt8-2bp* seed coats. Since PAs and PA monomers are flavonoids, their deficiency in *tt8-2bp* seed coats suggests disrupted flavonoid biosynthesis. Lignin, whose precursors are derived from the phenylpropanoid pathway, was present in the thickened oi1 cell walls of both *tt8-2bp* and wild-type seed coats. Because the flavonoid and phenylpropanoid pathways overlap, changes in one may influence the other. Alterations in seed coat components such as PAs, lignin, and other cell wall materials can impact seed coat hydrophobicity, permeability, and mechanical strength (Debeaujon *et al*., 2007). However, the precise effects of the *tt8-2bp* mutation on the metabolic pathways governing lignin formation, PA biosynthesis, and cell wall synthesis remain to be elucidated.

### Metabolomic characterization of flavonoids, lignin precursors, and cell wall polysaccharides in developing and mature non-embryonic tissues

Flavonoids, phenylpropanoids, and cell wall polysaccharides are essential for seed development and function, contributing to plant defense, stress responses, and structural strength. The flavonoid and phenylpropanoid pathways produced PAs, PA monomers, and lignin precursors. PAs and lignin are the two most abundant aromatic polymers in plants. Lignin and cell wall polysaccharides are particularly important for the mechanical strength and hydrophobicity of seed coats. Using metabolite annotations from the KEGG database and earlier studies, we mapped DE flavonoids and phenylpropanoids to their biosynthetic pathways. We also identified two major cell wall metabolites in our metabolomic analyses.

To characterize the tissue localization of LC-MS-identified DE metabolites, we performed spatial metabolomics by MALDI-MSI on 27 DAP seed cryosections at 25×25 μm^2^ resolution (Supplementary Fig. S9A; see the Materials and methods). Here, MALDI-MSI measured relative abundances of various molecular features in wild-type and *tt8-2bp* seed samples. By overlaying MALDI-MSI and brightfield images, we mapped molecular features to specific seed compartments (embryo, endosperm, ii layers, and thickened oi1 cell wall; Supplementary Fig. S9B). MALDI-MSI identifies molecular features by mass-to-charge ratios (*m/z*) but cannot distinguish mass isomers [e.g. C_6_H_12_O_9_S (*m/z* 259.0129) may represent D-galactose 6-sulfate, D-glucose 6-sulfate, or 6-deoxy-6-sulfo-D-gluconate). Thus, signal intensities detected by MALDI-MSI reflect the combined abundance of all ionized molecules sharing the same molecular formula and *m/z*. Since all seed sections were analyzed simultaneously to minimize any batch effect, we can compare relative abundances of a molecular feature in *tt8-2bp* versus wild-type seed compartments.

**Fig. 8.**
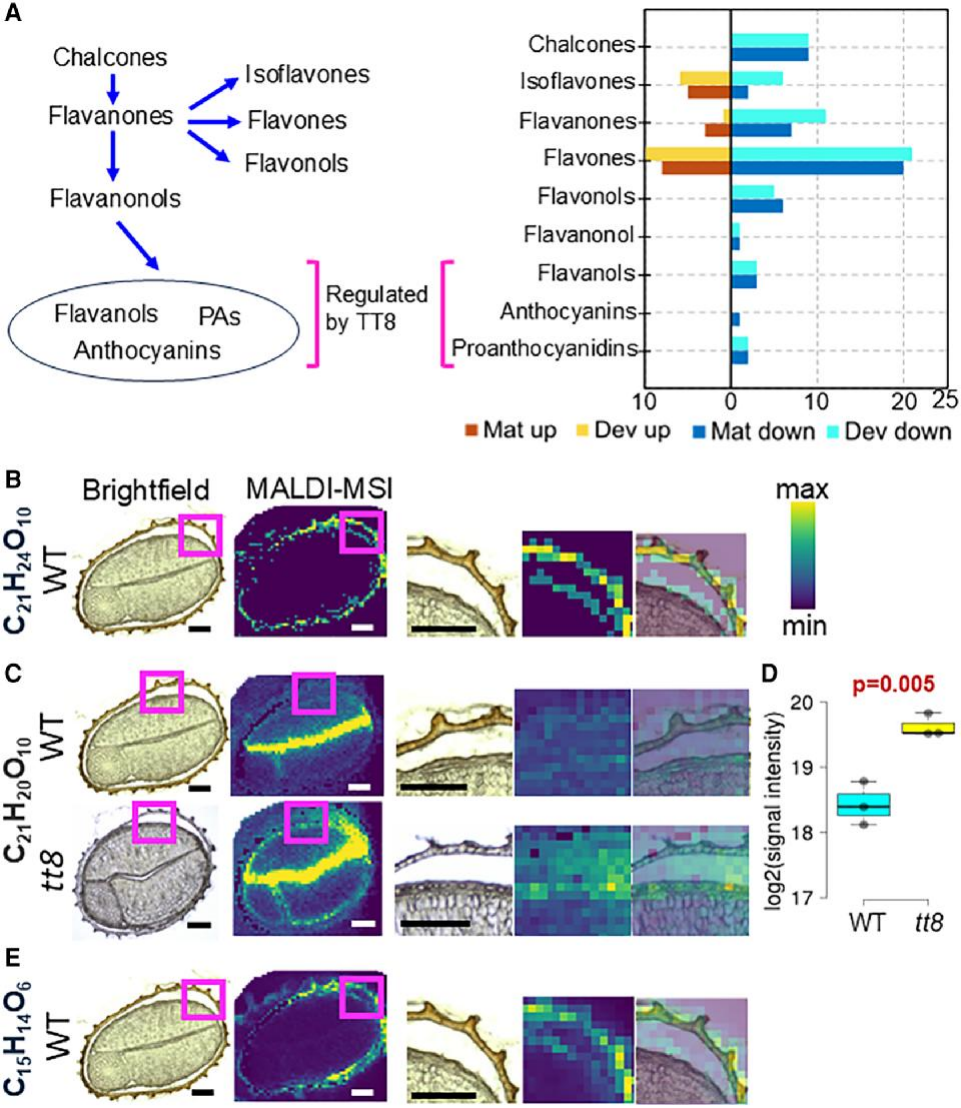
Different types of flavonoids among the differentially expressed (DE) metabolites in developing and mature non-embryonic tissues. (A) A diagram and a bar chart show the main types and numbers of flavonoids among the DE metabolites (*tt8-2bp* versus the wild type) in developing (Dev) and mature (Mat) non-embryonic seed tissues. Among the DE flavonoids, biosynthesis of flavonols, anthocyanins, and PAs is directly regulated by TT8. (B, C, E) Spatial metabolomic (MALDI-MSI) images of molecular features representing flavonoids and their mass isomers in developing (27 DAP) wild-type (WT) and *tt8-2bp* (*tt8*) seeds. The WT brightfield image is repeated in Supplementary Fig. S9C. The molecular features shown in (B), (C), and (E) have the molecular formulae C_21_H_24_O_10_ (*m/z* 435.1297), C_21_H_20_O_10_ (*m/z* 431.0984), and C_15_H_14_O_6_ (*m/z* 289.0718), respectively, all detected under the negative ionization mode. In (B), (C), and (E), from left to right are the brightfield micrographs, MALDI-MSI heatmaps, and magnified images of the selected regions (magenta boxes) of WT and *tt8* seed cryosections. The magnified images from left to right are brightfield image, MALDI-MSI heatmaps, and overlays of the former two. The MALDI-MSI images of WT and *tt8* of the same molecular feature are shown in the same color scale. Scale bars=200 µm. In *tt8* seed sections, C_21_H_24_O_10_ and C_15_H_14_O_6_ signals were below the detection threshold and thus no image is shown. C_21_H_24_O_10_ has one candidate molecule, phlorizin, which is a chalcone, and decreased in developing and mature *tt8* NETs based on LC-MS data. C_21_H_20_O_10_ has seven candidate molecules, of which six are flavonoids. Candidate molecules of C_21_H_20_O_10_ include isovitexin which is a flavone and is increased in developing and mature *tt8* NETs based on LC-MS data. C_15_H_14_O_6_ has 13 candidate molecules, of which seven are flavonoids. Candidate molecules of C_15_H_14_O_6_ include catechin and epicatechin which are flavanols and are decreased in developing and mature *tt8* NETs based on LC-MS data. (D) A boxplot of log2-transformed average ion signal intensities of C_21_H_20_O_10_ detected in three WT and *tt8* seed sections, respectively. The *P*-value was calculated by a two-tailed Student’s *t*-test comparing the log2-transformed average ion signal intensities of *tt8* versus the WT. Gray dots represent signals of different seeds.

The flavonoid biosynthesis pathway in plants is well characterized (Debeaujon *et al*., 2007; Liu *et al*., 2021; Zhuang *et al*., 2023) and the synthesis of nine main flavonoid classes is summarized in a simplified diagram in Fig. 8A. The pathway starts with phenylalanine, which is converted to chalcones—flavonoids and key precursors for most other flavonoid classes. Chalcones are converted into flavanones, which in turn can be converted by different enzymes into isoflavones, flavones, flavonols, or flavanonols. Flavanonols serve as precursors for biosynthesis of flavanols (PA monomers), PAs, and anthocyanins. TT8 forms a transcription factor complex with TTG1 and TT2, and activates several key enzymes involved in the synthesis of flavanols, PAs, and anthocyanins (Xu *et al*., 2015). To investigate the impact of the *tt8-2bp* mutation on flavonoid biosynthesis in NETs, we summarized DE flavonoids across all nine classes described before (Fig. 8A; Supplementary Table S14). Only some isoflavones, flavanones, and flavones upstream of TT8 regulation were up-regulated in developing and mature *tt8-2bp* NETs. In contrast, there were flavonoids in all nine flavonoid classes down-regulated in both developing and mature *tt8-2bp* NETs. The only exception is peonidin-3-*O*-α-arabinoside, the only DE anthocyanin, which was down-regulated exclusively in mature *tt8-2bp* NETs. As for flavanols and PAs, three flavanols (catechin, epicatechin, and afzelechin) and two PAs (PA B1 and PA B2) were down-regulated in both developing and mature *tt8-2bp* NETs. Catechin and epicatechin are monomers for PA B1 and B2 biosynthesis (Xie and Dixon, 2005).

To validate the DE flavonoids identified by LC-MS and determine their localization in NETs, we searched MALDI-MSI data for DE flavonoids upstream of TT8 regulation, as well as DE anthocyanins, flavanols, and PAs. Due to differences in ionization and identification methods, not all DE flavonoids identified by LC-MS were detected by MALDI-MSI. MALDI-MSI detected two molecular features representing a chalcone (phlorizin) and a flavone (isovitexin), both upstream of TT8 regulation. LC-MS results showed markedly reduced phlorizin abundances in developing and mature *tt8-2bp* NETs (0.004- and 0.01-fold of those in wild-type NETs). Consistently. MALDI-MSI detected a molecular feature C_21_H_24_O_10_ (*m/z* 435.1297) representing only phlorizin with strong ion signals in the thickened oi1 cell wall and areas where ii layers are located (above endosperm) in all three wild-type seed sections, and C_21_H_24_O_10_ ion signals were below the detectable threshold in *tt8-2bp* seed sections (Fig. 8B; Supplementary Fig. S9B). LC-MS results showed increased isovitexin abundances in developing and mature *tt8-2bp* NETs (12.5- and 1.7-fold of those in wild-type NETs). MALDI-MSI detected a molecular feature C_21_H_20_O_10_ (*m/z* 431.0984) representing seven mass isomers, comprising isovitexin, five other flavonoids, and emodin 8-glucoside (Fig. 8C; Supplementary Fig. S9B). In all wild-type and *tt8-2bp* seed sections, ion signals of C_21_H_20_O_10_ were strongest in embryos and weaker in areas where endosperm and seed coat cells were located. The average ion signal intensities of C_21_H_20_O_10_ in the NETs were significantly higher in *tt8-2bp* versus wild-type seed sections (*P*<0.05, Fig. 8D). Additionally, MALDI-MSI identified two molecular features representing two flavanols (catechin and epicatechin) and one PA (PA B2), respectively, whose biosynthesis is TT8 dependent. Based on LC-MS, catechin and epicatechin had drastically decreased abundances in developing and mature *tt8-2bp* NETs (<0.09-fold of those in wild-type NETs). PA B2 had drastically decreased abundance in developing *tt8-2bp* NETs (0.0004-fold of that in developing wild-type NETs). Molecular features detected in MALDI-MSI representing catechin, epicatechin, and PA B2 showed very similar ion signal patterns where the ion signals were strong in wild-type seed coats (the thickened oi1 cell wall and ii layers) but below the detectable threshold in *tt8-2bp* seed sections (Fig. 8E; Supplementary Fig. S9C, D). The molecular feature C_15_H_14_O_6_ (*m/z* 289.0718) represented 13 mass isomers including catechin, epicatechin, five other flavonoids, and five non-flavonoid metabolites, and the molecular feature C_30_H_26_O_12_ (*m/z* 577.1351) represented four different PAs comprising PA B2, PA B4, PA B5, and epicatechin-(4β→8)-ent-epicatechin. In summary, MALDI-MSI corroborated LC-MS findings for five DE flavonoids, and showed that phlorizin, catechin, epicatechin, and PA B2 are localized primarily in seed coats. The localization patterns of PA B2 and its monomers in wild-type seeds were consistent with histological analyses (summarized in Supplementary Table S15).

**Fig. 9.**
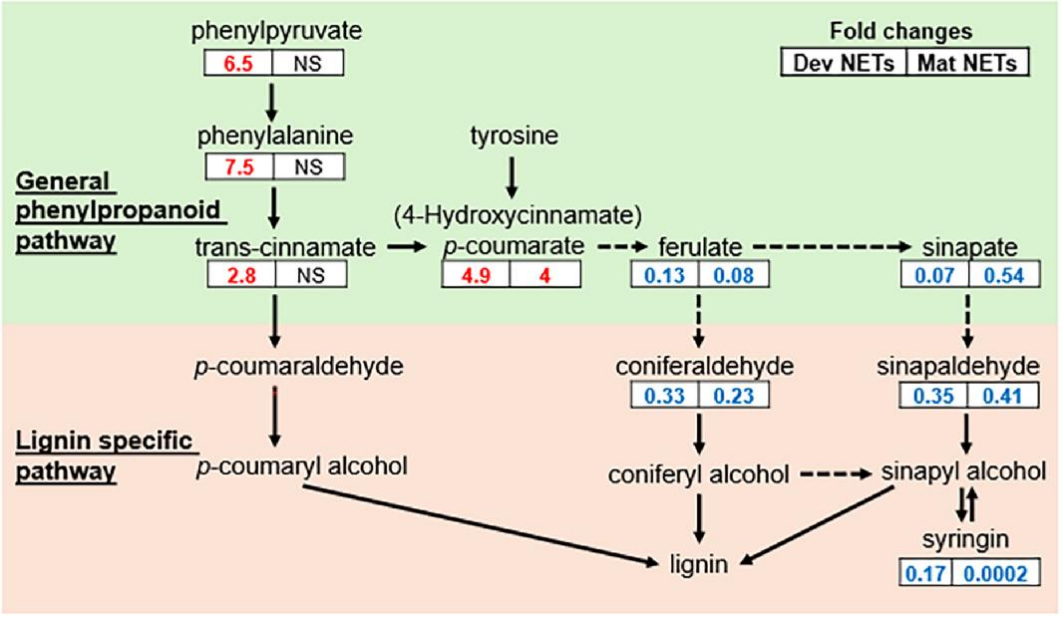
Differentially expressed (DE) metabolites (*tt8-2bp* versus wild-type seed tissues) detected by LC-MS metabolomic analysis belonging to the phenylpropanoid pathway with important roles in lignin biosynthesis. Fold changes of DE metabolites in developing (Dev) and mature (Mat) non-embryonic tissues (NETs) are listed below each metabolite (left and right), respectively. Red letters indicate up-regulation and blue letters indicate down-regulation in *tt8-2bp* NETs. For example, phenylpyruvate had significantly increased abundances in developing *tt8-2bp* NETs (6.5-fold of those in wild-type NETs). NS, not significant. Dashed lines indicate multiple enzymatic reactions. After Xie *et al*. (2018).

Phenylpropanoids are a diverse group of secondary metabolites produced via the phenylpropanoid pathway, playing key roles in plant defense, stress response, and structural support. Lignin, a complex aromatic polymer in secondary cell walls, contributes to structural strength and hydrophobicity of plant tissues. Lignin is formed through the oxidative coupling of three monolignols, coniferyl alcohol, sinapyl alcohol, and *p*-coumaryl alcohol, which are synthesized by the phenylpropanoid pathway (Steck *et al*., 2022; Seidi *et al*., 2025). In developing and mature *tt8-2bp* and wild-type NETs, nine DE metabolites essential for monolignol synthesis were identified, including three monolignol precursors coniferaldehyde, sinapaldehyde, and syringin (Fig. 9; Supplementary Table S16). The three monoligonol precursors, along with ferulate and sinapate, showed decreased abundances in *tt8-2bp* NETs, while four DE metabolites synthesized earlier in the phenylpropanoid pathway had increased abundances in developing and/or mature *tt8-2bp* NETs (Fig. 9; Supplementary Table S16). MALDI-MSI analysis revealed inconsistent ion signal patterns of the DE phenylpropanoids identified by LC-MS in seed cross-sections under positive versus negative ion modes, preventing meaningful interpretation. Additionally, LC-MS did not detect monolignols, potentially because most were converted to oligolignols at the developmental stages analyzed, thus a conclusion cannot be reached regarding the changes in lignin formation in *tt8-2bp* versus wild-type NETs.

Beyond flavonoids and phenylpropanoids, we searched LC-MS and MALDI-MSI data for cell wall metabolites in developing and mature NETs of *tt8-2bp* and the wild type. Histological staining revealed markedly increased oi1 cell wall thickness and reduced nutrient storage of *tt8-2bp* and wild-type seed coats by 27 DAP, indicating cell wall metabolites as main chemical components of seed coats after 27 DAP. Xylose and galacturonate, the main components of hemicellulose and pectin, respectively, were found in LC-MS and/or MALDI-MSI data. Hemicellulose and pectin are the main components of primary and secondary cell walls. Based on LC-MS, xylose had decreased abundances in developing and mature *tt8-2bp* NETs (0.2- and 0.1-fold those in wild-type NETs, Supplementary Table S12), indicating reduced cell wall polysaccharides in seed coats and endosperm. Xylose was not detected in MALDI-MSI. Based on LC-MS, a molecular feature C_6_H_10_O_7_ (*m/z* 193.03533) representing galacturonate and/or 5-keto-D-gluconate had no significant differences in developing or mature NETs (Supplementary Tables S5, S9), and the ambiguity of the identity of C_6_H_10_O_7_ was due to low metabolite abundances. MALDI-MSI data showed similar results. Ion signals of some sugar acids and sugar acid derivatives (C_6_H_10_O_7_) and digalacturonate and glycyrrhizin (C_12_H_18_O_13_) were found in seed coat compartments of the wild type and *tt8-2bp*. The molecular feature C_6_H_10_O_7_ represents 20 sugar acids and sugar acid derivatives, many of which were pectin precursors or pectin degradation products such as D-galacturonate and D-fructuronate. C_6_H_10_O_7_ ion signals were localized to the thickened oi1 cell wall and cells above the embryo (Supplementary Fig. S9E), consistent with the pectin locations in seed coats based on histological staining. Digalacturonate and glycyrrhizin were the only two candidate molecules identified for C_12_H_18_O_13_. C_12_H_18_O_13_ ion signals had the same localizations as those of C_6_H_10_O_7_ (Supplementary Fig. S9G). The C_12_H_18_O_13_ ion signals localized to thickened oi1 cell wall in wild-type and *tt8-2bp* samples were most probably from digalacturonate since digalacturonate is a pectin degradation product whereas glycyrrhizin is a triterpene compound stored in vacuoles (Kato *et al*., 2022). Neither C_6_H_10_O_7_ nor C_12_H_18_O_13_ had significant differences in abundances in *tt8-2bp* versus the wild type (Supplementary Fig. S9F, H), suggesting that pectin biosynthesis was likely to be unaffected in *tt8-2bp* seed coats (summarized in Supplementary Table S15).

### Solid-state NMR showed reduced aromatic compounds and cell wall polysaccharides in oi1 cell walls of mature *tt8-2bp* seed coats

**Fig. 10.**
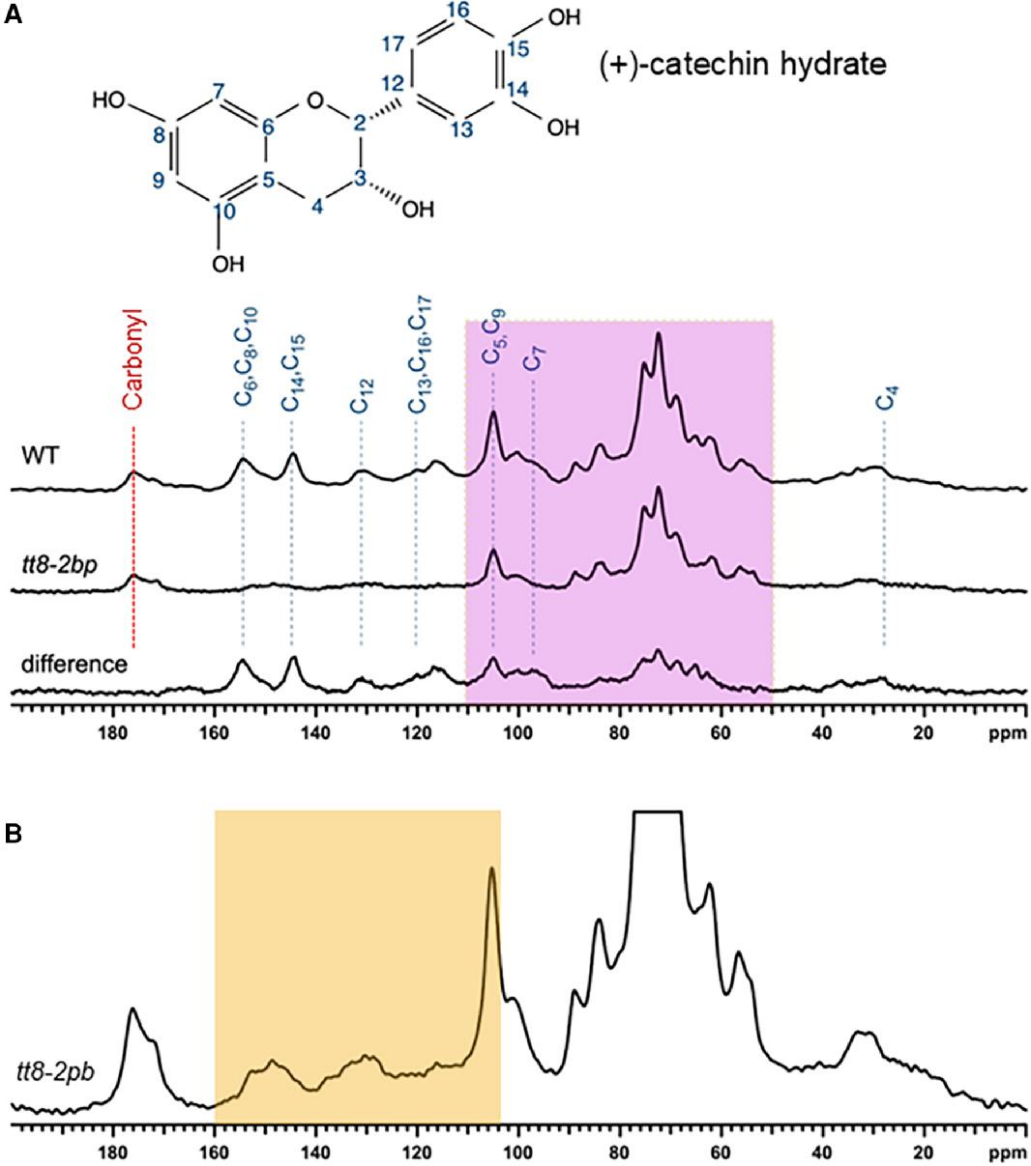
^13^C CP-MAS spectra of wild-type (WT) and *tt8-2bp* mature outer seed coat show drastic reduction in aromatic compounds in *tt8-2bp* seed coats. (A) Spectra of the WT and *tt8-2bp* after scaling peak intensities to the 176 ppm peak, representing the carbonyl group of D-galacturonate and homogalacturanan, the main components of pectin. The difference spectrum was obtained by subtracting the peak intensities of the *tt8-2bp* spectrum from the WT spectrum. (+)-Catechin hydrate was identified as the main aromatic compound contributing to the differences between the WT and *tt8-2bp* at 50–110 ppm. The molecular structure is shown above the spectra where the numbers correspond to the C number labeled in the spectra below. The pink box highlights peak groups at 50–110 ppm representing chemical groups of cellulose, hemicellulose, and other general carbohydrates. (B) Zoomed-in *tt8-2bp* spectrum emphasizes peak groups at 104–160 ppm (orange box) where chemical groups of aromatic compounds including lignin and PAs are located.

Histological staining and metabolomic analyses revealed a significant reduction of PAs and PA monomers in both the ii1 and thickened oi1 cell walls of 27 DAP *tt8-2bp* seed coats, without significant impacts on cell wall pectin. Additionally, *tt8-2bp* NETs showed a notable decrease in xylose based on LC-MS, suggesting a potential reduction of cell wall polysaccharides in *tt8-2bp* seed coats. In both wild-type and *tt8-2bp* mature seeds, the lignified and thickened oi1 cell wall eventually forms the protective outermost seed coat (magenta brackets in Supplementary Fig. S10A). To compare chemical compositions of wild-type versus *tt8-2bp* outermost seed coats, we performed ssNMR analysis which quantifies large polymers such as lignin, PAs, cellulose, and pectin (see the Materials and methods). The outermost seed coats of mature seeds were dissected manually and 57 mg of fully hydrated outermost seed coats of the wild type and *tt8-2bp* were used for ssNMR. For quantitative comparison, spectra of the wild type and *tt8-2bp* were scaled to the 176 ppm peak corresponding to the carbonyl groups in D-galacturonate and homogalacturonan (Table 1; Fig. 10A), the main pectin components. This is because pectin levels appeared similar between the wild type and *tt8-2bp* based on prior histology and metabolomic results (Supplementary Figs S6C, S9E–H; Supplementary Table S15).

**Table 1.** Assignment of peaks in ^13^C CP/MAS NMR spectra of pennycress outer seed coat

Chemical shift (ppm)	Assignment
176	Carbonyl of D-galacturonate and homogalacturanan
154	C_6_, C_8_, C_10_
144	C_14_, C_15_
130	C_12_
120	C_13_, C_16_, C_17_
105	C_5_, C_9_
96	C_7_
98–158	Aromatic compounds
50–110	Cellulose, hemicellulose, and general carbohydrates C_2_, C_3_, C_5_
29	C_4_

A difference spectrum (Fig. 10A) revealed that wild-type seed coats had higher peak intensities for aromatic compounds (98–158 ppm) and polysaccharides (50–110 ppm) compared with *tt8-2bp*. Annotated peaks are listed in Table 1. After aligning the wild-type and difference spectra of aromatic compounds, a close match was found with the known NMR spectrum of (+)-catechin hydrate (Martínez-Richa and Joseph-Nathan, 2003). (+)-Catechin hydrate is a hydrated form of catechin, a major PA monomer (Yu *et al*., 2022). We noted that the C5, C9, and C7 peaks of (+)-catechin hydrate overlapped with the range of peak groups representing polysaccharides (Fig. 10A; Table 1), suggesting that the peak intensities of C5, C9, and C7 peaks probably reflected chemical groups in (+)-catechin hydrate and other polysaccharides. In the *tt8-2bp* spectrum, peaks representing aromatic compounds were much lower but detectable upon magnification (Fig. 10B). One prominent peak (105 ppm) of the *tt8-2bp* spectrum coincides with the C5, C9 peak of (+)-catechin hydrate, suggesting that the *tt8-2bp* peak was partially from cell wall polysaccharides. In prior analyses, lignin was detected in thickened oi1 cell walls of 27 DAP wild-type and *tt8-2bp* seed coats (Fig. 4C) whereas PAs and PA monomers were undetectable by histological staining or MALDI-MSI in *tt8-2bp* seed coats (Supplementary Figs S9D, S10A). Additionally, some isoflavones, flavanones, and flavones had increased abundances in mature *tt8-2bp* NETs, with some DE flavonoids potentially localized to the thickened oi1 cell wall (Fig. 8). Thus, the low intensity peaks representing aromatic compounds in the *tt8-2bp* spectrum, excluding those overlapping with polysaccharides, probably represent lignin and some monomeric flavonoids (Fig. 10B). As for polysaccharides (50–110 ppm), the wild-type spectrum had higher peak intensities than the *tt8-2bp* spectrum (Fig. 10A), suggesting that wild-type seed coats had more cell wall polysaccharides such as cellulose and hemicellulose. This is also consistent with reduced xylose in *tt8-2bp* NETs measured by LC-MS. Additionally, the spectra of the wild type and *tt8-2bp* showed very weak signal from very-long-chain fatty acids, the main component of cuticle and waxes, which are represented by a sharp peak at ∼30 ppm (Fig. 10A). This suggests that cuticle and waxes are not major components of mature pennycress outermost seed coats. In summary, ssNMR analysis shows that PAs are the predominant aromatic polymers in wild-type seed coats whereas lignin is the main aromatic polymers in *tt8-2bp* seed coats, which also had markedly reduced PAs, PA monomers, and cell wall polysaccharides.

### *tt8-2bp* seeds had increased embryo to non-embryonic tissue ratios

**Fig. 11.**
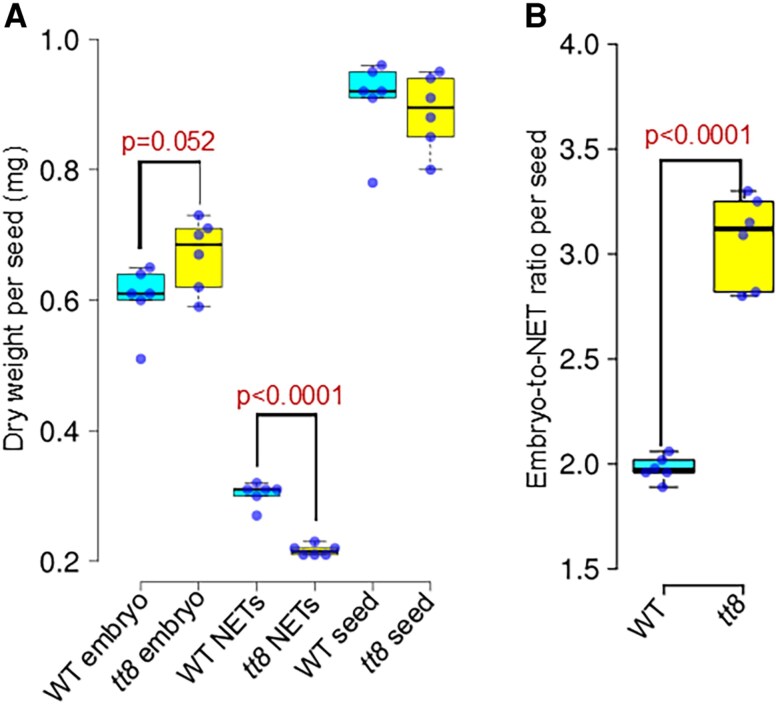
Mature *tt8-2bp* (*tt8*) seeds have significantly reduced seed coat dry weights and increased embryo dry weights and embryo-to-NET ratios compared with wild-type (WT) seeds. All measurements were done for six biological replicates of 75 seeds per biological replicate. (A) Average dry weights of embryo, non-embryonic tissues (NETs), and whole seeds (seed) per seed after dehydration. (D) Ratios of embryo-to-NETs (dry weights) calculated based on values in (A). *P*-values are calculated by two-tailed Student’s *t*-tests with a threshold of *P*≤0.05.

Histological staining, metabolomic, and ssNMR analyses revealed that the amounts of aromatic compounds and cell wall polysaccharides were significantly reduced in the developing and mature *tt8-2bp* seed coats and NETs compared with those of the wild type. Because PAs are the main aromatic polymers in wild-type seed coats and cell wall polysaccharides are the major components of primary and secondary cell walls, we hypothesized that nutrient partitioning between embryos and NETs might differ between the two genotypes. To test this, we measured the dry weights of embryos, NETs, and whole seeds harvested from six mature wild-type and *tt8-2bp* plants (see the Materials and methods). Mature *tt8-2bp* seeds had significantly lower NET dry weights and higher embryo dry weights than wild-type seeds, while whole seed dry weights were similar between the two genotypes (Fig. 11A). This indicates altered total nutrient partitioning between embryos and NETs in *tt8-2bp* seeds. Calculating embryo-to-NET dry weight ratios, we found that *tt8-2bp* seeds had significantly higher ratios (3:1) than wild-type seeds (2:1) (Fig. 11B). Since no significant difference was found in whole seed dry weights, we can assume that the total nutrients measured by dry weights fixed in mature seeds including storage compounds (e.g. starch, lipids, and proteins) and structural components (e.g. cell wall fibers) were about equal for the wild type and *tt8-2bp*. The embryo-to-seed coat ratios were 2:1 and 3:1 for wild-type and *tt8-2bp* seeds, respectively, meaning that 75% of total seed nutrients were fixed in *tt8-2bp* embryos versus 66% in wild-type embryos. Thus, *tt8-2bp* mature embryos fixed, on average, 9% more nutrients measured by dry weights than wild-type embryos.

## Discussion

Earlier research on *tt2* and *tt8* KO mutants in *Brassicaceae* oil crops, including pennycress, has shown that these mutations reduce seed dormancy and fiber contents while increasing oil and protein contents, making *TT2* and *TT8* promising targets for pennycress domestication. Nonetheless, how these mutations affect pennycress seed development and other agronomic traits remains unclear. In this study, we conducted detailed anatomical and histochemical analyses of developing and mature pennycress seeds. We observed no significant differences in the development rate or anatomy of *tt8-2bp* embryos and endosperms compared with those of the wild type. However, *tt8-2bp* pennycress seeds exhibited drastic alterations in seed coat anatomy and chemical compositions, most notably a deficiency in PAs and PA monomers. This PA and PA monomer deficiency correlated with increased seed coat permeability, increased imbibition rates, and altered seed aging, all of which are traits important for agronomy and affected by seed coat anatomy and chemical compositions. To further investigate, we performed metabolite profiling of developing and mature NETs of *tt8-2bp* and wild-type seeds. LC-MS metabolomics revealed significant reductions in flavonoids (including PAs and PA monomers), phenylpropanoids (including lignin monomer precursors), and the cell wall polysaccharide xylose in *tt8-2bp* NETs. Spatial metabolomics confirmed a marked decrease in several flavonoids, including PAs and the PA monomer catechin, particularly in the thickened oi1 cell wall of *tt8-2bp* seed coats. To further verify the changes in mature *tt8-2bp* seed coats, the chemical compositions of the outermost protective coat (mainly the thickened oi1 cell wall) of mature wild-type and *tt8-2bp* seeds were analyzed via ssNMR. Results showed that PAs were the major aromatic polymers in wild-type coats while *tt8-2bp* coats had sharply reduced aromatic compounds and cell wall polysaccharides, corroborating the metabolomics data. Additionally, mature *tt8-2bp* seeds had reduced NET dry weights and increased embryo dry weights, indicating altered nutrient allocation between the NETs and embryo during *tt8-2bp* seed development. Earlier studies have analyzed the metabolite and transcript profiles of wild-type pennycress embryos (Tsogtbaatar *et al*., 2015; Johnston *et al*., 2022), but our study is the first to provide an in-depth analysis on the seed coat metabolite profiles of wild-type and *tt8* KO mutant seeds.

### Pennycress seed coat anatomy and chemical compositions

Seed development and anatomy are well characterized in Arabidopsis and *B. napus* (Wan *et al*., 2002; Haughn and Chaudhury, 2005; Gao *et al*., 2022). The embryo and endosperm development of pennycress underwent similar anatomical and morphological changes to Arabidopsis and *B. napus* (Fig. 2), but notable differences exist in seed coat anatomy and chemical compositions. Pennycress seed coats have three ii and three oi layers (Fig. 3A), while Arabidopsis has three ii and two oi layers (Haughn and Chaudhury, 2005; Huang *et al*., 2023). In *B. napus*, the number of ii layers decreases from 6–8 at plant flowering to 2–3 at the torpedo stage of embryo development, with four oi layers present throughout seed development (Wan *et al*., 2002). Despite these anatomical differences, all three species accumulate PAs in the ii1 layer and form thickened cell walls outside oi1 (Figs 3B, 4A; Windsor *et al*., 2000; Haughn and Chaudhury, 2005; Zhang *et al*., 2022), suggesting that PA accumulation in ii1 and formation of thickened oi1 cell wall are likely to be evolutionarily conserved. However, only pennycress wild-type seeds were shown to accumulate PAs in the thickened oi1 cell wall (Figs 3B, 8E, 10A; Supplementary Fig. S9D; Supplementary Table S15). PA accumulation in oi layers has not been reported for Arabidopsis or *B. napus*. One *B. napus* study reported PAs in the oi1 (palisade) layer, but the histological evidence supporting this conclusion was insufficient due to low-resolution micrographs (Qu *et al*., 2013). Additionally, mucilage production in the oi2 layer is unique to Arabidopsis seed coats among the three species compared (Jaimie *et al*., 2005; Haughn and Western, 2012). Pennycress Spring32 seed coats did not accumulate or extrude mucilage (Supplementary Fig. S6B, C), consistent with previous findings (Viudes *et al*., 2021). The synthesis and secretion of seed mucilage is a complex process regulated by >100 genes (Viudes *et al*., 2021). *MUCILAGE-MODIFIED* (*MUM*) genes, first identified in Arabidopsis, are among the most extensively studied regulators of mucilage secretion. Many genes modulating mucilage synthesis and secretion, such as *TTG1*, *MUM2*, and *MUM4*, are pleiotropic and function in various cellular processes across different tissues (Western *et al*., 2004; Macquet *et al*., 2007b). Since seed mucilage is primarily composed of pectin and other polysaccharides such as cellulose, which are major components of the plant cell wall, many genes involved in mucilage secretion also participate in cell wall formation. Consequently, gene expression alone does not reliably predict mucilage secretion in seeds of different plant species.

Seed coat lignin and PAs are major aromatic polymers contributing to seed fiber contents. Conventional fiber quantification methods do not distinguish between lignin and PAs (Steck *et al*., 2022). However, differentiating these components is crucial for targeted improvements in seed quality and nutrient profiles. Comparative analyses have revealed species-specific aromatic polymer compositions: grape outer seed coats contain both lignin and PAs, chokeberry coats contain only PAs, and raspberry coats contain only lignin (Steck *et al*., 2022). In wild-type pennycress seeds, both PAs and lignin were detected in the seed coats. PAs were distributed throughout the thickened oi1 cell wall, and oxidized PAs contributed to the brown coloration of mature seed coats (Fig. 4B; Supplementary Figs S2A, S9D; Supplementary Table S15). SsNMR identified (+)-catechin hydrate, a key PA precursor, as the predominant aromatic compound in the outermost seed coat of wild-type mature seeds (Fig. 10A), indicating that PAs are the main aromatic polymers and fiber source of pennycress wild-type seeds. Minimal phenolic compounds were detected by ssNMR in PA-deficient *tt8-2bp* seeds in the outermost seed coat (Fig. 10). Lignin appeared to localize in small regions of the thickened oi1 cell walls of both wild-type and *tt8-2bp* seed coats (Fig. 4C). Reduced lignin monomer precursors were detected by LC-MS in *tt8-2bp* NETs (Fig. 9), suggesting reduced lignin formation or altered lignin composition. Taken together, our study suggests that reducing PA accumulation is likely to be a more effective strategy for lowering seed fiber contents than targeting lignin biosynthesis in pennycress seeds, supporting *TT2* and *TT8* as promising targets for pennycress domestication.

### Altered *tt8-2bp* seed traits and their agronomic implications

Mature *tt8-2bp* seeds exhibited increased seed coat permeability to water-soluble molecules (Fig. 5A, B), faster imbibition rates (Fig. 6A), and accelerated aging under high temperature and humidity (Fig. 6B) compared with wild-type seeds. These altered seed traits can be at least partially attributed to reduced seed coat hydrophobicity and altered permeability, which are regulated by the seed coat cuticle and PAs in both Arabidopsis and beans (Ranathunge *et al*., 2010; Demonsais *et al*., 2020; Upretee *et al*., 2024). The seed coat cuticle and PAs form a barrier that protects seeds from pathogens, water, and oxygen, thereby preventing damage, limiting oxidative stress, and delaying seed aging (Debeaujon *et al*., 2007; Mira *et al*., 2011; Upretee *et al*., 2024). In Arabidopsis, ii1 cells accumulate PAs and are closely associated with the endosperm via a seed coat cuticle layer (Loubery *et al*., 2018; Demonsais *et al*., 2020). Arabidopsis *tt* KO mutant seeds have increased seed coat permeability due to PA deficiency in ii1 cells and impaired seed coat cuticle (Loubery *et al*., 2018; Demonsais *et al*., 2020; Niñoles *et al*., 2023). Pennycress wild-type and *tt8-2bp* seeds share the same anatomy as Arabidopsis, with ii1 cells tightly associated with the endosperm through a cuticle layer (Fig. 5C). However, whether the cuticle lipid composition was altered in *tt8-2bp* seed coats was unclear. It has been reported that different cuticle lipids affect permeability differently (de Silva *et al*., 2021). Potential changes in *tt8-2bp* seed cuticle could have contributed to the altered seed traits, but further analyses using TEM and GC are required to assess potential changes in *tt8-2bp* cuticle structure and lipid composition (e.g. changes in porosity or suberin content of the cuticle layer).

Altered seed traits in *tt8* KO mutants have important implications for seed viability, germination, and storage. Enhanced imbibition rates in *tt8* KO seeds can promote faster germination under favorable moisture and temperature conditions. However, increased seed coat permeability in *tt8* KO mutants heightens susceptibility to soil contaminants, such as chromium and residual herbicides, which can permeate across the seed coat and impair embryo viability. An Arabidopsis study on *tt7* KO mutant seeds showed that increased *tt7* seed coat permeability was associated with decreased seed longevity (Niñoles *et al*., 2023). While wild-type pennycress seed germination and final yields were unaffected by corn herbicides based on a field study (Bishop and Nelson, 2019), *tt8* and *tt2* KO mutant seeds can be affected due to increased seed coat permeability. Nonetheless, reduced field persistence of *tt8* and *tt2* KO seeds is advantageous for pennycress as an intermediate crop, as it minimizes weed pressure in subsequent corn or soybean rotations. A field study showed that after 1 month of burial in well-drained soil, *tt8* KO seed germination drops below 5%, whereas wild-type seeds maintain nearly 60% germination after 2 years (Wesley *et al*., 2025, Preprint). Additionally, the increased permeability of *tt8* and *tt2* KO seeds can affect their response to chemical treatments before planting. Extended exposure to chemicals such as fungicides can damage embryos and reduce germination. For instance, fungicide treatment decreased *tt8* KO seedling establishment by 15–20%, though final yields were unaffected compared with fungicide-treated wild-type seeds (Koirala *et al*., 2023). Therefore, chemical treatments should be optimized on small seed batches to prevent significant reduction in germination and seedling establishment. Altered seed coat properties of *tt8* and *tt2* KO mutants can also impact seed longevity during storage. The optimal storage condition for *tt8* KO seeds is at 2 °C and 22% humidity, contrasting with wild-type seeds, which store best at 22 °C and 54% humidity (Brandhorst *et al*., 2024). In this study, high humidity (54%) at room temperature may have accelerated aging in *tt8* KO seeds, probably due to oxidative stress caused by increased seed moisture contents during storage. Testing *tt8* and *tt2* KO seed storage at room temperature and low humidity (≤20%) could help reduce storage costs if seed viability is maintained.

### Altered nutrient partitioning between embryo and seed coat of *tt* mutants

In *tt8-2bp* seeds, reduced seed coat aromatic polymers (primarily PA) and cell wall polysaccharides were associated with higher embryo dry weights and lower NET dry weights compared with wild-type seeds (Fig. 11). The increase in *tt8-2bp* embryo dry weights can be partly attributed to elevated oil contents, as *tt8-2bp* seeds had ∼10% more total seed oil than wild-type seeds (Gautam *et al*., 2026). This is consistent with the established role of *TT8* as a negative regulator of fatty acid biosynthesis in Arabidopsis (Chen *et al*., 2014). Similar changes in seed storage nutrients have been observed in *tt* KO mutants of other *Brassicaceae* species. Arabidopsis *ttg1* mutants showed increased embryo dry weights and elevated starch, protein, and fatty acid levels (Chen *et al*., 2015). Disruption of *TT* genes in *Camelina sativa* L. and *B. napus* led to higher total fatty acids and storage lipids in mature seeds (Zhai *et al*., 2020; Cai *et al*., 2024; Li *et al*., 2024). Collectively, these findings suggest that *TT* genes have a conserved function in regulating seed coat flavonoid synthesis and seed storage nutrient accumulation, particularly fatty acids and lipids, within the *Brassicaceae* family.

In *tt* KO mutants, increased fatty acid and oil accumulation is closely linked to altered nutrient partitioning between seed coats and embryos. Seed oil contents show positive correlations with sucrose, starch, and storage protein levels in embryos (Chen *et al*., 2015; Zhai *et al*., 2020; Apriyanto *et al*., 2022), but are negatively correlated with seed coat thickness and cell wall components such as lignin, cellulose, and hemicellulose (Miao *et al*., 2019; Zhang *et al*., 2022). Supporting this, yellow-seed pennycress mutants with elevated oil content exhibit reduced seed coat thickness (Griffiths *et al*., 2025). In *B. napus*, *TT8* expression correlates positively with seed coat contents (seed coat weight to total seed weight ratios) and negatively with oil contents (Zhang *et al*., 2022). Furthermore, KO mutations in *TTG1*, *TT2*, and *TT8* in Arabidopsis and *B. napus* down-regulate flavonoid biosynthesis genes and up-regulate fatty acid biosynthesis genes (Chen *et al*., 2012, 2014, 2015; Li *et al*., 2024). In summary, the TTG1–TT2–TT8 transcription factor complex regulates the expression of genes involved in flavonoid and fatty acid biosynthesis, thereby modulating nutrient allocation between NETs and embryos. Therefore, *TTG1*, *TT2*, and *TT8* represent promising targets for enhancing seed quality and nutrient contents in pennycress and other *Brassicaceae* oil crops.

In future studies, it will be important to dissect the molecular mechanisms by which *TT8*, together with *TT2* and *TTG1*, regulates pennycress seed development to further guide seed trait improvement. In our study, *tt8-2bp* and wild-type pennycress seeds showed similar embryo and seed areas at 7–23 DAP whereas *tt8-2bp* embryos and seeds were significantly smaller at 27 DAP (Supplementary Fig. S2B, C), suggesting changes in late stages of seed maturation (e.g. seed filling and/or dehydration). This could be caused by direct effects of *tt8* KO mutations on seed development or indirect consequences of seed coat defects (reduced PAs and cell wall polysaccharides, Figs 8, 10) accelerating seed dehydration. Further investigation is needed to distinguish these possibilities and determine whether the reduced *tt8-2bp* seed and embryo areas at 27 DAP are related to the increased embryo dry weights of mature *tt8-2bp* seeds (Fig. 11). Characterizing *TT8* expression patterns in developing pennycress seeds is essential to understand how it regulates seed development. Although not examined in this study, pennycress *TT8* gene expression patterns may be similar to those of Arabidopsis *TT8*. This is because pennycress and Arabidopsis wild-type seeds both accumulate PAs in the innermost seed coat ii layer and their *tt8* mutants have similar seed coat defects, namely PA deficiency and increased permeability (Figs 3, 5A; Debeaujon *et al*., 2000). Since Arabidopsis *TT8* is expressed in the PA-accumulating seed coat cells (Xu *et al*., 2013), pennycress *TT8* is probably also expressed in the PA-accumulating ii1 and oi1 layers. Tissue-specific transcriptomic profiling of developing pennycress seeds (*tt8* KO and wild type) would characterize *TT8* expression patterns and help construct gene regulatory networks linking *TT8* and other key regulators in seed development. It has been shown that seed coats not only provide physical protection for embryos and endosperm but also play an important role in regulating their growth and development, as reviewed by Pankaj *et al*. (2024) and Figueiredo and Sharma (2026). In Arabidopsis seeds, *TT8* expression in seed coats regulates seed fatty acid contents and triploid block during interploidy crosses through maternal control (Chen *et al*., 2014; Zumajo-Cardona *et al*., 2023a). For example, if the mother plant is a homozygous *tt8* KO mutant, the F_1_ seeds will have higher fatty acid contents compared with wild-type seeds regardless of the genetic background of the father plant (Chen *et al*., 2014). Similarly, F_1_ seed lethality caused by triploid block during interploidy crosses can be fully suppressed as long as the mother plant is a homozygous *tt8* KO mutant (Zumajo-Cardona *et al*., 2023a). Reciprocal crosses between pennycress *tt8* KO mutants and wild-type plants can help assess how maternal *TT8* expression regulates seed coat chemical compositions and permeability, embryo and seed sizes, endosperm development (e.g. nucleus numbers and timing of cellularization), and seed nutrient contents.

In this study, we showed that a knockout mutation in pennycress *TT8* resulted in reduced seed coat fiber and NET dry weights, alongside increased embryo dry weights, without detectable anatomical defects in the embryo or endosperm. Although *tt8-2bp* seeds exhibited higher imbibition rates, which may promote faster germination, their increased seed coat permeability could accelerate aging during storage and heighten susceptibility to chemical or environmental damage. These potential drawbacks can be mitigated by optimizing seed treatment and field management practices. Further field studies are necessary to assess *tt8-2bp* seed germination and seedling establishment under abiotic stresses such as residual herbicides, drought, salinity, and waterlogging.

## Supplementary Material

erag078_Supplementary_Data

## Data Availability

All MALDI-MSI images and corresponding brightfield images are publicly available on the METASPACE repository at https://metaspace2020.org/project/XinXin-2025 (Ding *et al*., 2025).

## Supplementary data

The following supplementary data are available at *JXB* online.

Table S1. Consecutive incubation steps for seed sample dehydration, clearing, and paraffin embedding.

Table S2. Consecutive incubation steps for seed paraffin section deparaffinization and rehydration before histological staining.

Table S3. RP and HILIC separation gradients for LC-MS metabolomics.

Table S4. Metabolite annotations and relative abundances detected under RP positive mode.

Table S5. Metabolite annotations and relative abundances detected under RP negative mode.

Table S6. Metabolite annotations and relative abundances detected under HILIC positive mode.

Table S7. Metabolite annotations and relative abundances detected under HILIC negative mode.

Table S8. ANOVA test results comparing relative metabolite abundances under RP positive mode of developing and mature *tt8-2bp* versus wild-type NETs.

Table S9. ANOVA test results comparing relative metabolite abundances under RP negative mode of developing and mature *tt8-2bp* versus wild-type NETs.

Table S10. ANOVA test results comparing relative metabolite abundances under HILIC positive mode of developing and mature *tt8-2bp* versus wild-type NETs.

Table S11. ANOVA test results comparing relative metabolite abundances under HILIC negative mode of developing and mature *tt8-2bp* versus wild-type NETs.

Table S12. Complete list of DE metabolites in NETs of *tt8-2bp* versus wild-type NETs.

Table S13. Two-tailed Student’s *t*-test results comparing areas of embryos and whole seeds (*tt8-2bp* versus wild type) measured from images taken using seed paraffin sections.

Table S14. DE metabolites belonging to flavonoids and annotations of flavonoid subclasses.

Table S15. Summary of integrated histochemical results of key metabolite classes in wild-type versus *tt8-2bp* seed coats.

Table S16. DE metabolites belonging to phenylpropanoids and whether they are involved in lignin synthesis.

Fig. S1. Timeline and representative images of Spring32 wild-type and *tt8-2bp* plant development from seedling establishment to seed maturity.

Fig. S2. Seed and embryo development in Spring32 wild-type and *tt8-2bp* mutants.

Fig. S3. Main seed tissues of a developing Spring32 wild-type pennycress seed.

Fig. S4. Additional micrographs of histological staining of wild-type and *tt8-2bp* embryo and endosperm show that the two seed tissues developed at a similar rate at 7–27 DAP.

Fig. S5. Paraffin sections of 15 DAP wild-type and *tt8-2bp* seeds unstained and stained with vanillin-HCl for detection of PAs.

Fig. S6. The thickened oi1 cell wall of the wild type and *tt8-2bp* was stained differently by alcian blue at 15–27 DAP ,and detection of mucilage and pectin in pennycress seeds.

Fig. S7. Seed coat cuticle staining of the micropyle and chalaza of 27 DAP wild-type and *tt8-2bp* seeds detected by auramine O staining.

Fig. S8. Principal component analysis plots of LC-MS data of NET samples analyzed under the RP and the HILIC positive and negative modes.

Fig. S9. Additional molecular features of interest identified in the spatial metabolomic analysis of small molecules and lipids in developing Spring32 wild-type and *tt8-2bp* seeds at 27 DAP.

Fig. S10. Cryosections of mature wild-type and *tt8-2bp* seeds showing the outermost seed coat used for the ssNMR analysis and the ^13^C CP/MAS spectra of *tt8-2p*.

## Abbreviations

DAP: days after pollination
HCl: hydrochloric acid
ii: inner integument
KO: knockout
LC-MS: liquid chromatography mass spectrometry
MALDI-MSI: matrix-assisted laser desorption ionization MS imaging
NETs: non-embryonic tissues
oi: outer integument
PA: proanthocyanidin
ssNMR: solid-state NMR
*tt8*: *transparent testa 8*
