# Supplementary material for: A pennycress transparent testa 8 knockout mutant has drastic changes in seed coat anatomy and chemical compositions
Source: J Exp Bot. 2026 Feb 13;77(7):2106–34. doi: 10.1093/jxb/erag078 (PMC13022552; doi:10.1093/jxb/erag078)
Supplement: erag078_Supplementary_Data [file erag078_supplementary_data.zip › jexbot316228-file001.pdf]

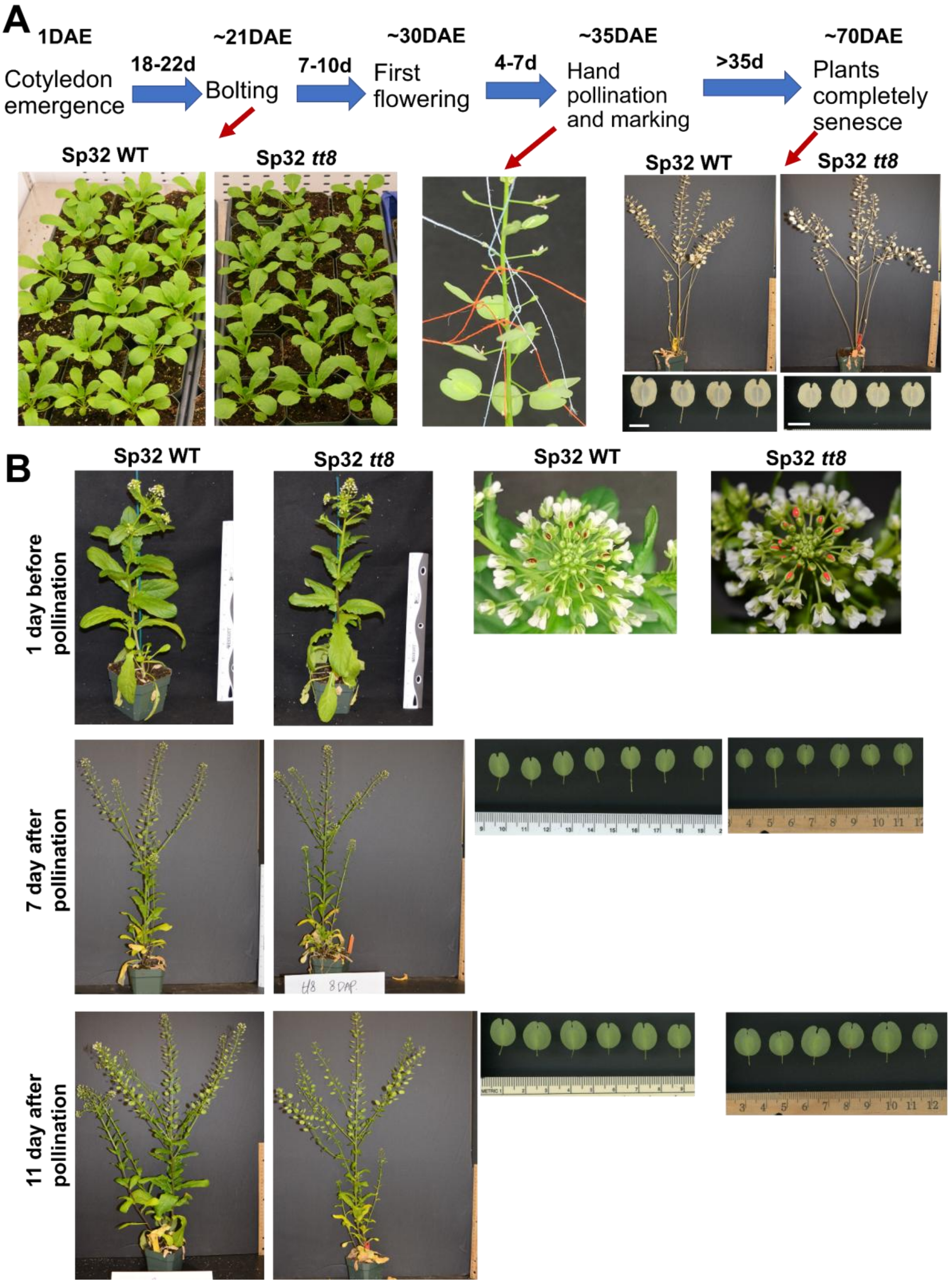
A
1DAE
Cotyledon emergence
18-22d
~21DAE
Bolting
7-10d
~30DAE
First flowering
4-7d
~35DAE
Hand pollination and marking
>35d
~70DAE
Plants completely senesce
Sp32 WT
Sp32 tt8
Sp32 WT
Sp32 tt8
B
Sp32 WT
Sp32 tt8
Sp32 WT
Sp32 tt8
1 day before pollination
7 day after pollination
11 day after pollination
tt8 8DAP

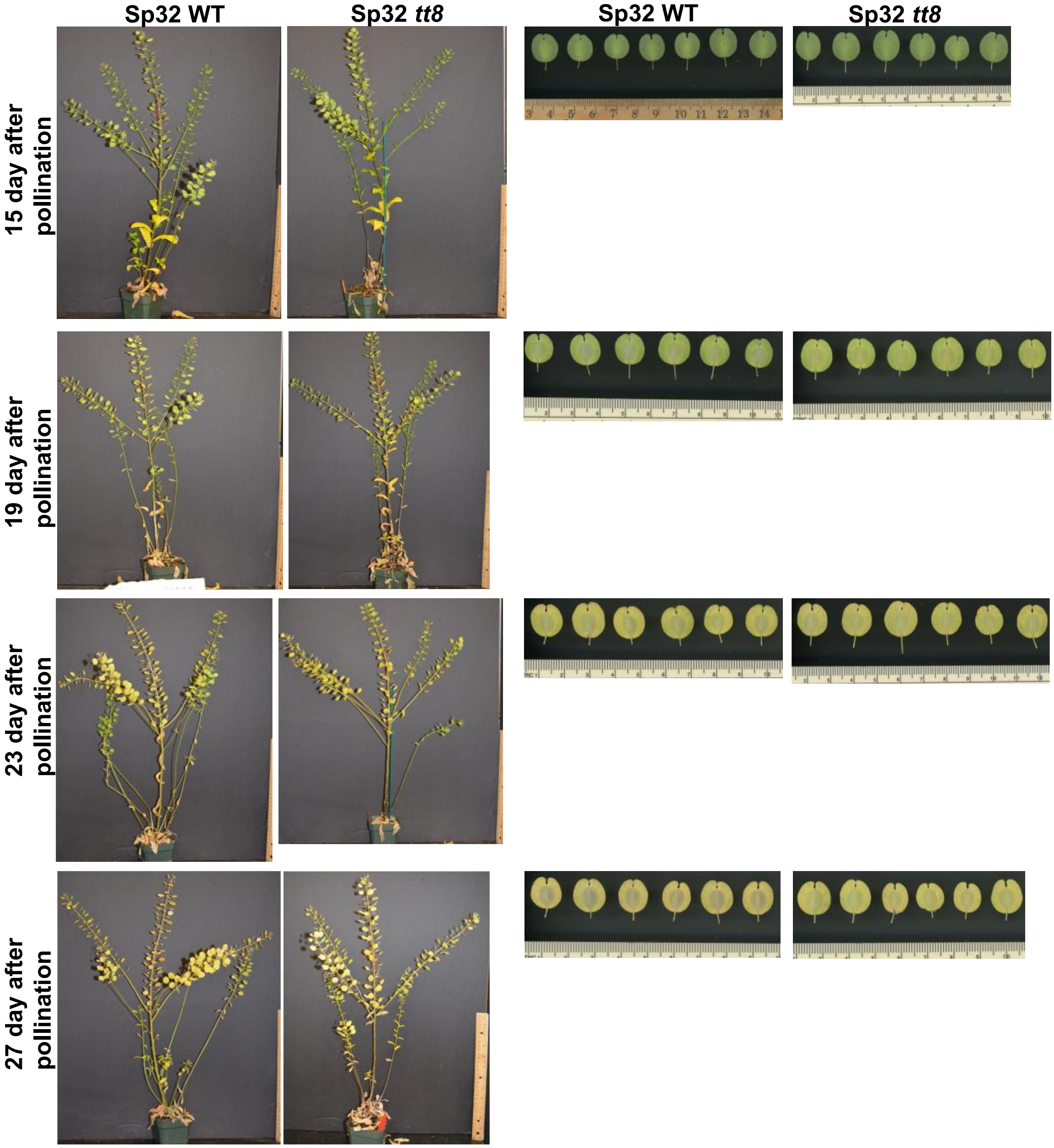

Supplementary Figure S1. Timeline and representative images of Spring32 wild-type and *tt8-2bp* plant development from seedling establishment to seed maturity. (A) Sterilized seeds were stratified for 2 days and germinated on filter paper. Germinated seedlings were planted and grown at 22 ºC, 50% humidity, under 200 μmol m^{-2} s^{-1} light. Fertilizer (Jack's Pro) was applied twice a week. Senesced plant pictures were taken on 41 day after pollination (DAP). DAE, day after emergence. Scale bars = 1 cm. (B) Representative images of Sp32 WT plants and siliques at different days after pollination when seed samples were harvested.

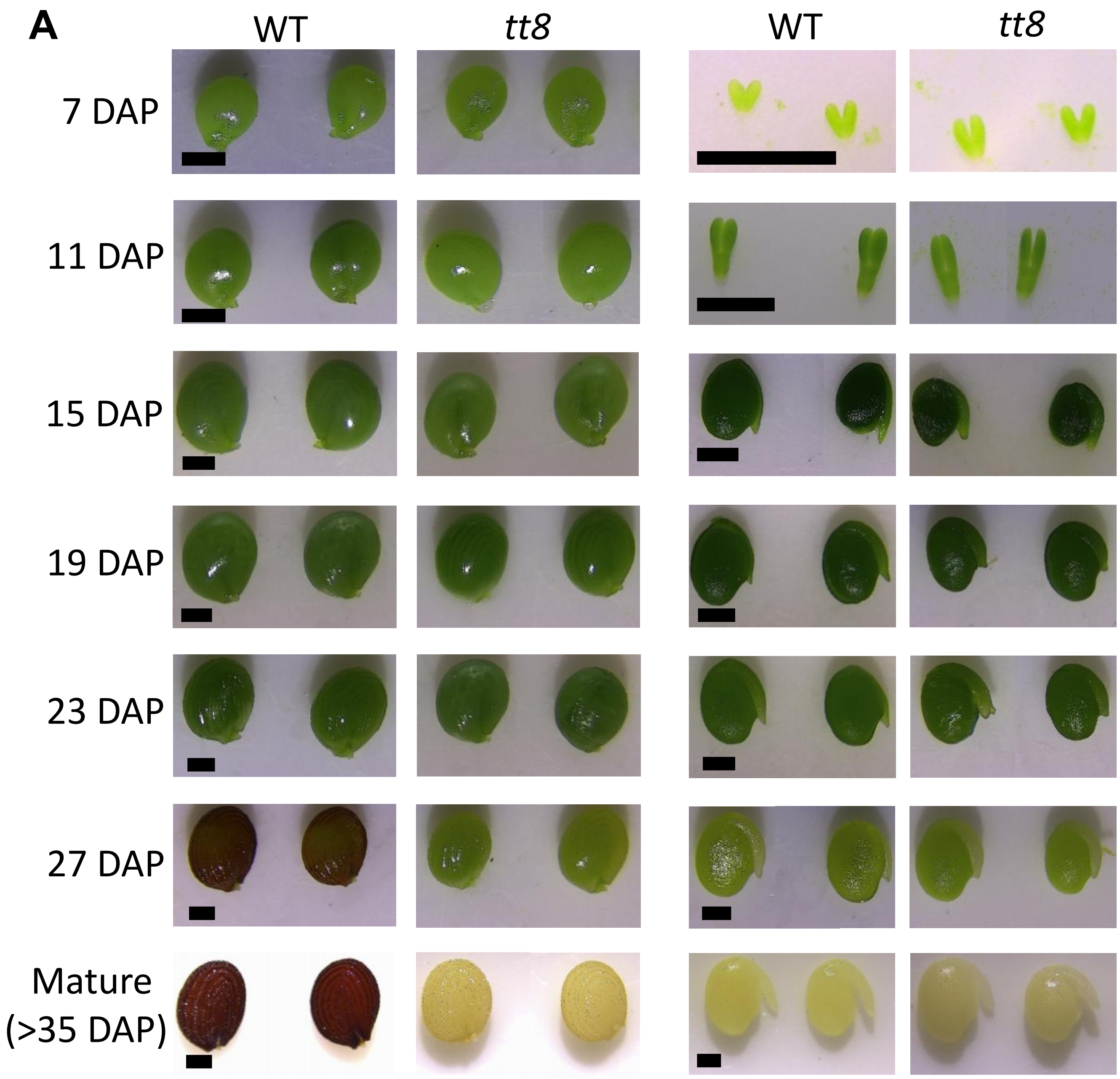
A
WT
tt8
WT
tt8
7 DAP
11 DAP
15 DAP
19 DAP
23 DAP
27 DAP
Mature
(>35 DAP)

B

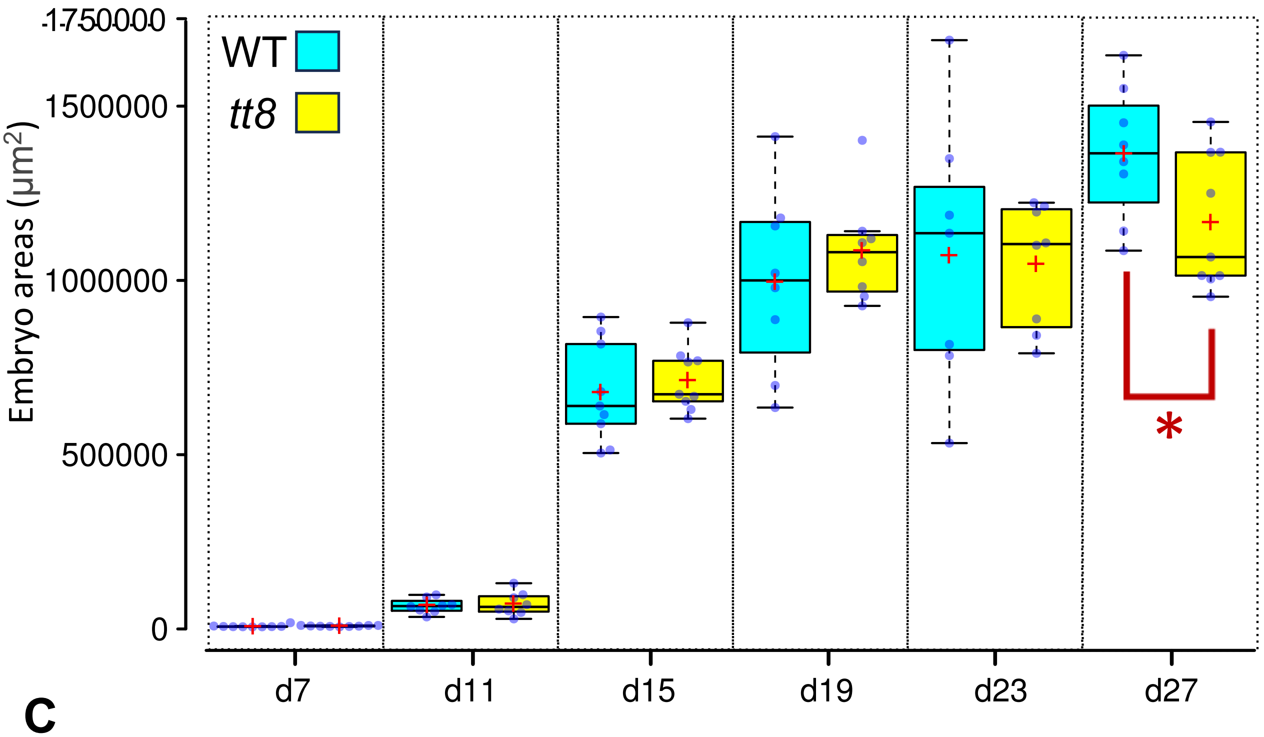

C

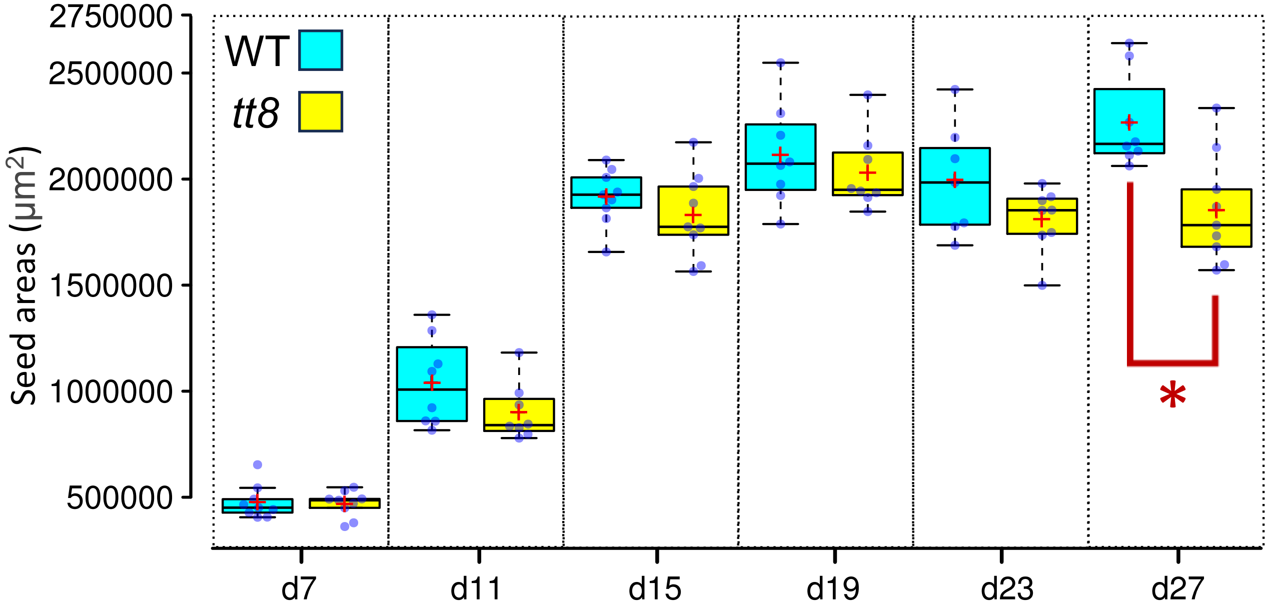

Supplementary Figure S2: Seed and embryo development in *Spring32* wild-type (WT) and *tt8-2bp* (*tt8*) mutants. (A) Representative images of seeds and isolated embryos from WT and *tt8* at the indicated days after pollination (DAP). Scale bars=500 μm. (B, C) Quantification of embryo (B) and seed (C) cross-section areas from similar images as in **Fig. 2A**. Data points (blue dots; n=8-9 seeds per genotype per DAP, one section per seed) represent individual measurements; red crosses indicate the mean. Statistical significance was determined by two-tailed Student's t-tests (p-value ≤ 0.05). Asterisks denote significant differences between WT and *tt8* at 27 DAP (d27).

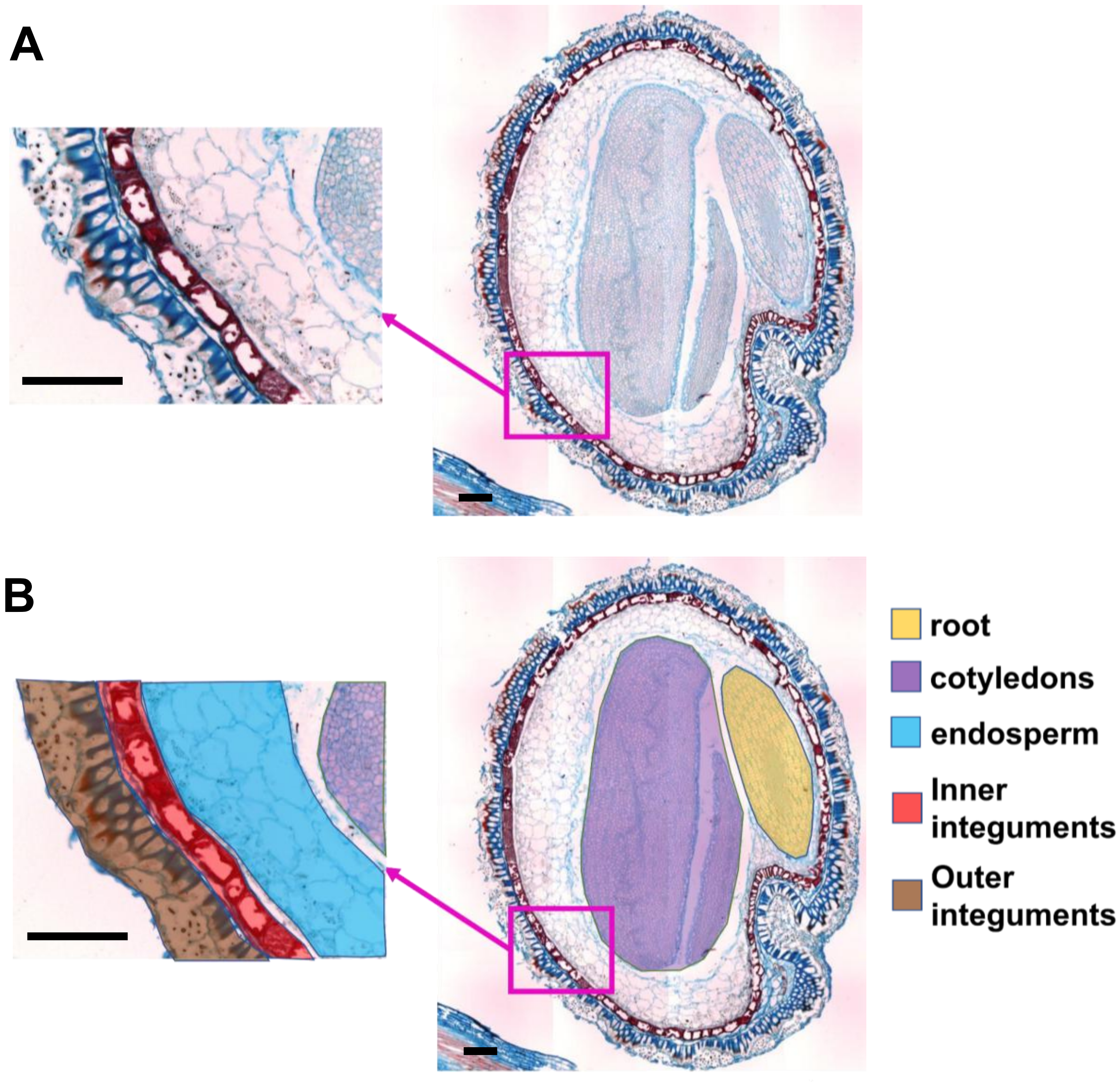

Supplementary Figure S3: Main seed tissues of a developing Spring32 wild-type pennycress seed. (A, B) A seed paraffin section of a WT seed at 15 DAP stained with safranin O and alcian blue and a blowup image focused on the seed coat cell layers. Scale bars = 100 µm.

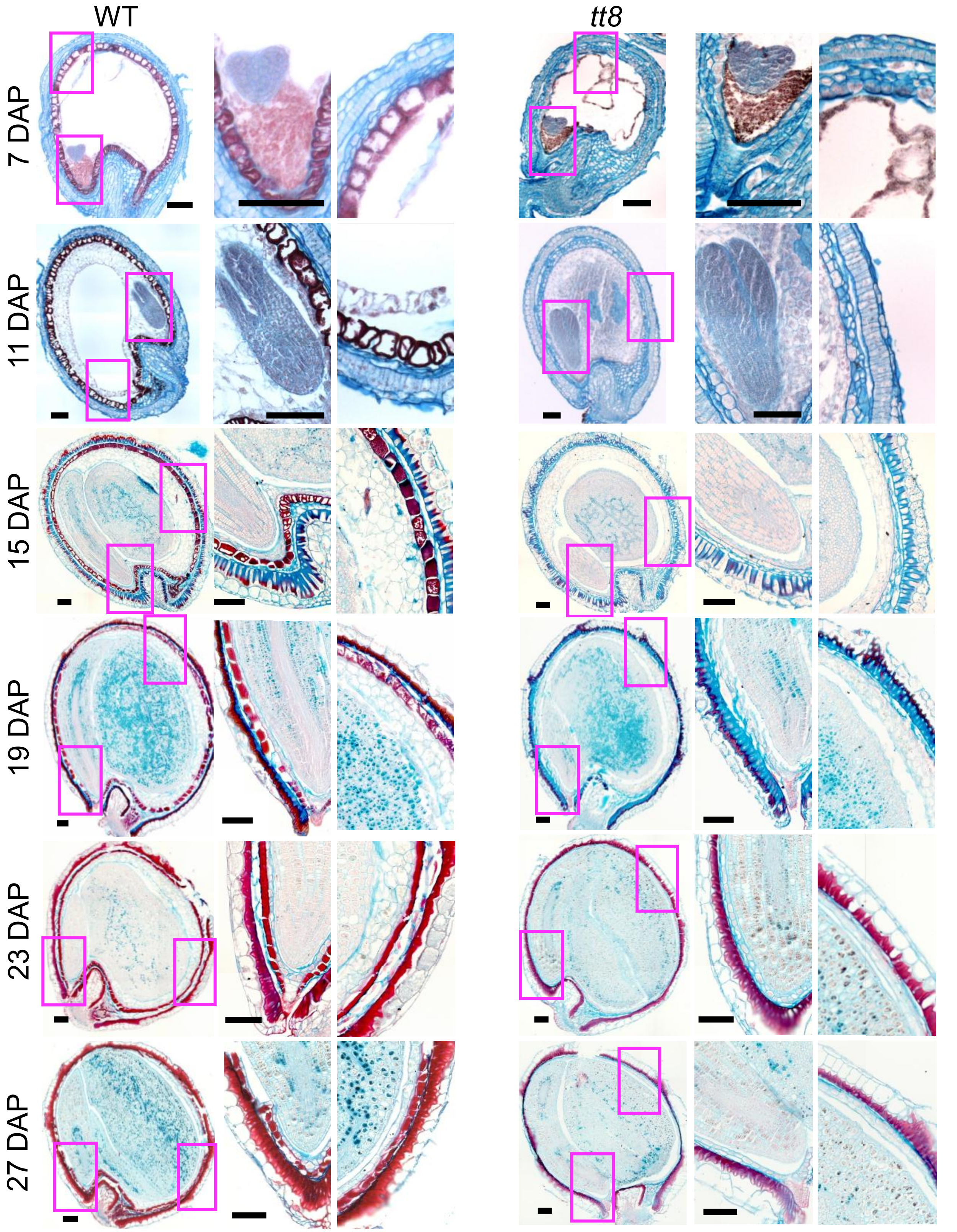

Supplementary Figure S4. Additional micrographs of histological staining of wild-type (WT) and *tt8-2bp* embryo and endosperm shows that the two seed tissues developed at a similar rate at 7-27 DAP. Seed sections were stained with safranin-O (stains secondary cell wall and nuclei red) and counter-stained with alcian blue (stains acidic polysaccharides of primary cell wall blue). Blowup images of shown on the right of each seed micrograph. Scale bars = 100 µm.

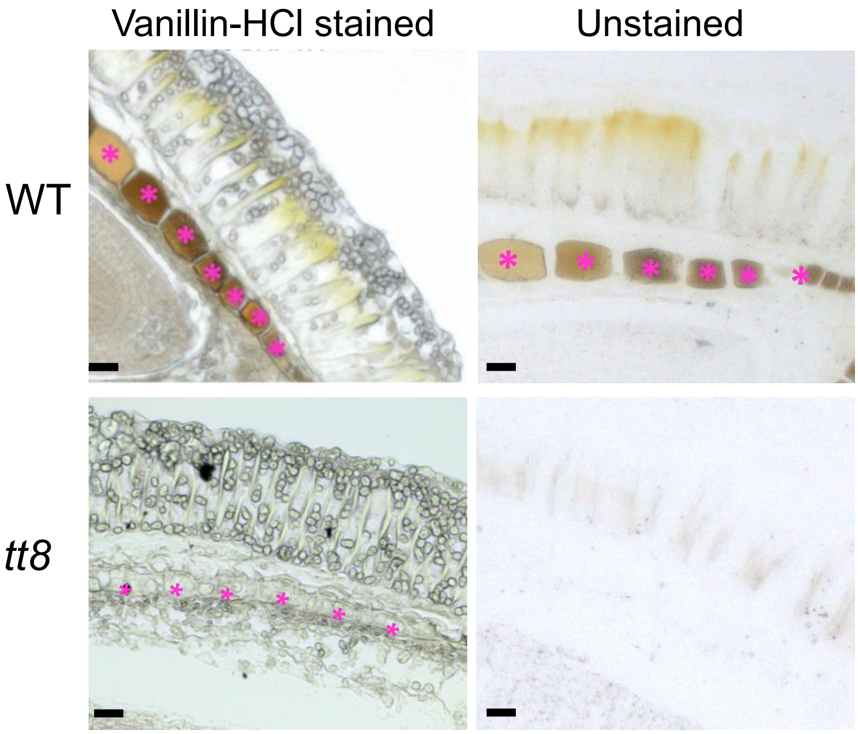

Supplementary Figure S5. Paraffin sections of 15 DAP wild-type (WT) and *tt8-2bp* seeds unstained and stained with vanillin-HCl for detection of PAs. Magenta asterisks mark the ii1 cells accumulating PAs. Note that in both WT and *tt8-2bp* sections, there was no difference in the color of the ii1 cells in the unstained *vs.* vanillin-HCl stained seed sections. Scale bars = 25 μm.

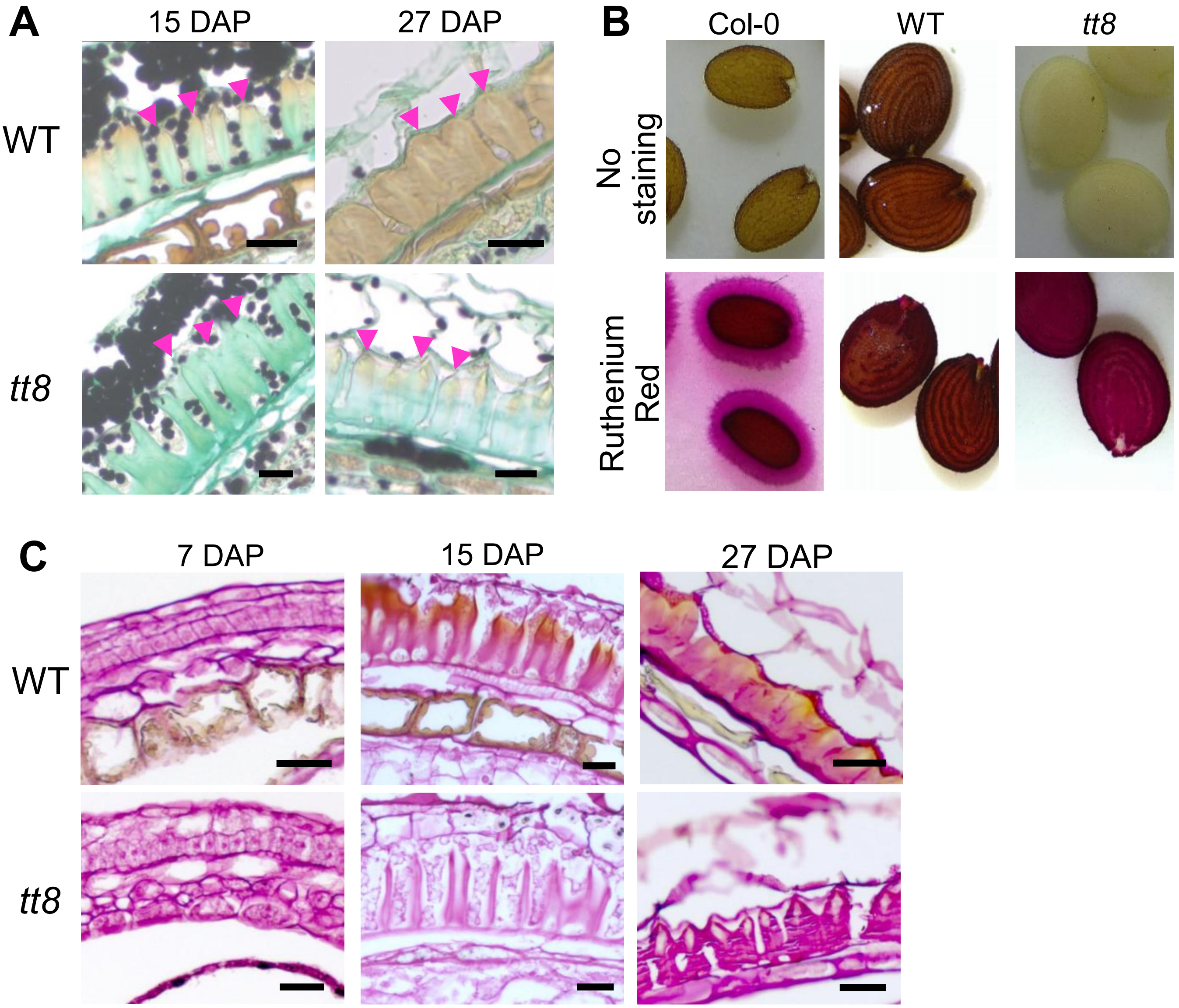

Supplementary Figure S6: The thickened oi1 cell wall of wild-type (WT) and *tt8-2bp* were stained differently by alcian blue at 15-27 DAP and detection of mucilage and pectin in pennycress seeds. (A) Seed sections of Sp32 WT and *tt8* at 15 and 27 DAP were stained with Alcian blue and Lugol's iodine which stain primary cell wall light turquoise and starch granules black, respectively. The magenta arrowheads point to the thickened oi1 cell walls which in WT seed coats appeared yellowish-brown at 27 DAP. (B) Mature seeds of Arabidopsis Col-0, pennycress Sp32 WT, and *tt8* with and without 0.1% ruthenium red staining after seed imbibition in 100mM EDTA (pH 8) to detect secretion of seed mucilage. (C) Pennycress seed paraffin sections stained with 0.1% ruthenium red. Scale bars = 25 µm.

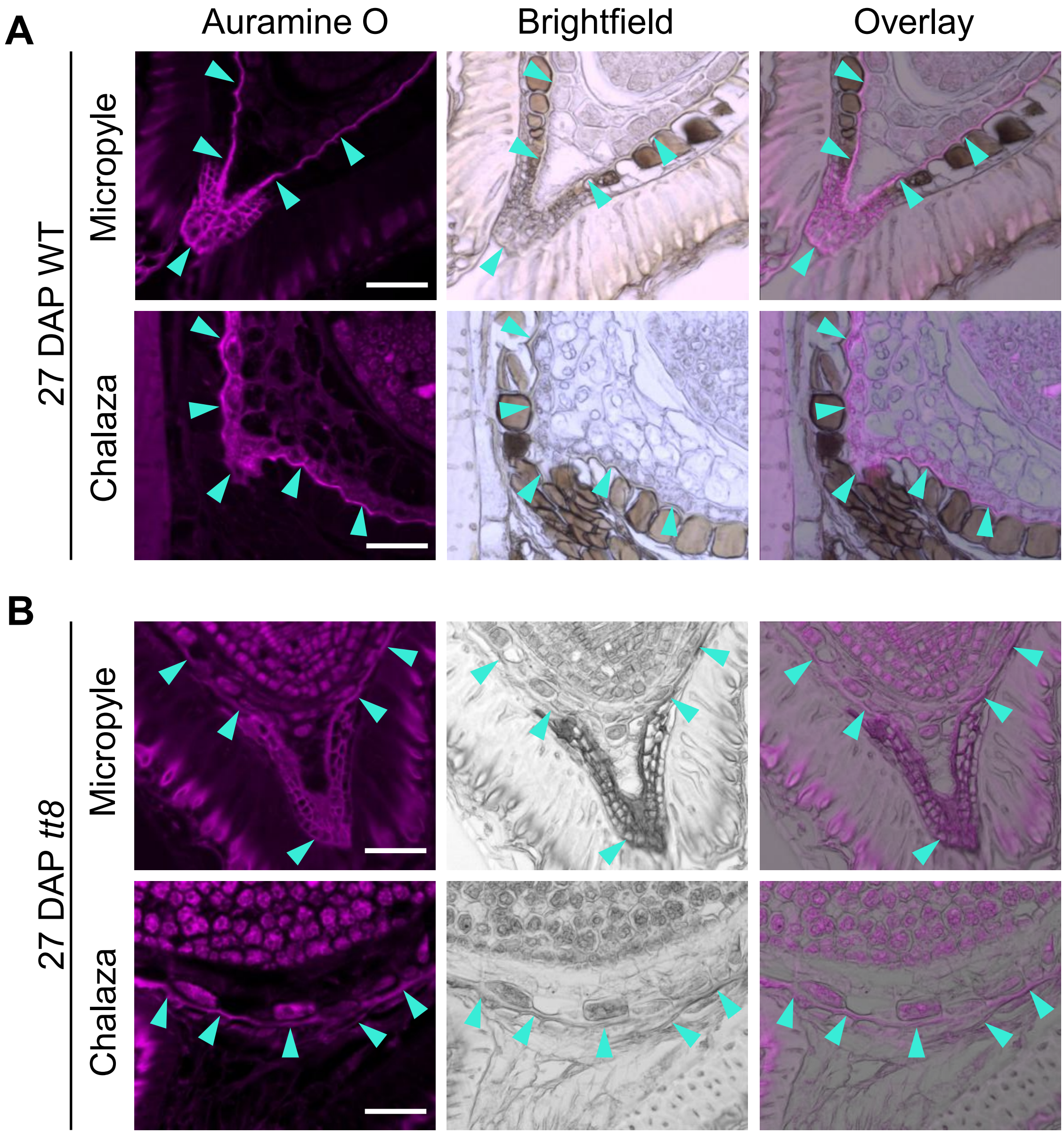

Supplementary Figure S7. Seed coat cuticle staining of the micropyle and chalaza of 27 DAP wild-type (WT) and *tt8-2bp* (*tt8*) seeds detected by Auramine O staining. Auramine O is a fluorescent dye which stains cuticle and lipids. Representative images of the micropyle and chalaza of WT and *tt8* seeds are shown in panel (A) and panel (B), respectively. Cyan arrowheads point to seed coat cuticle. Note that Auramine O also stained other compartments in WT and *tt8* seeds (*e.g.*, endosperm and embryo cells) with high lipid contents. Scale bars = 50 µm.

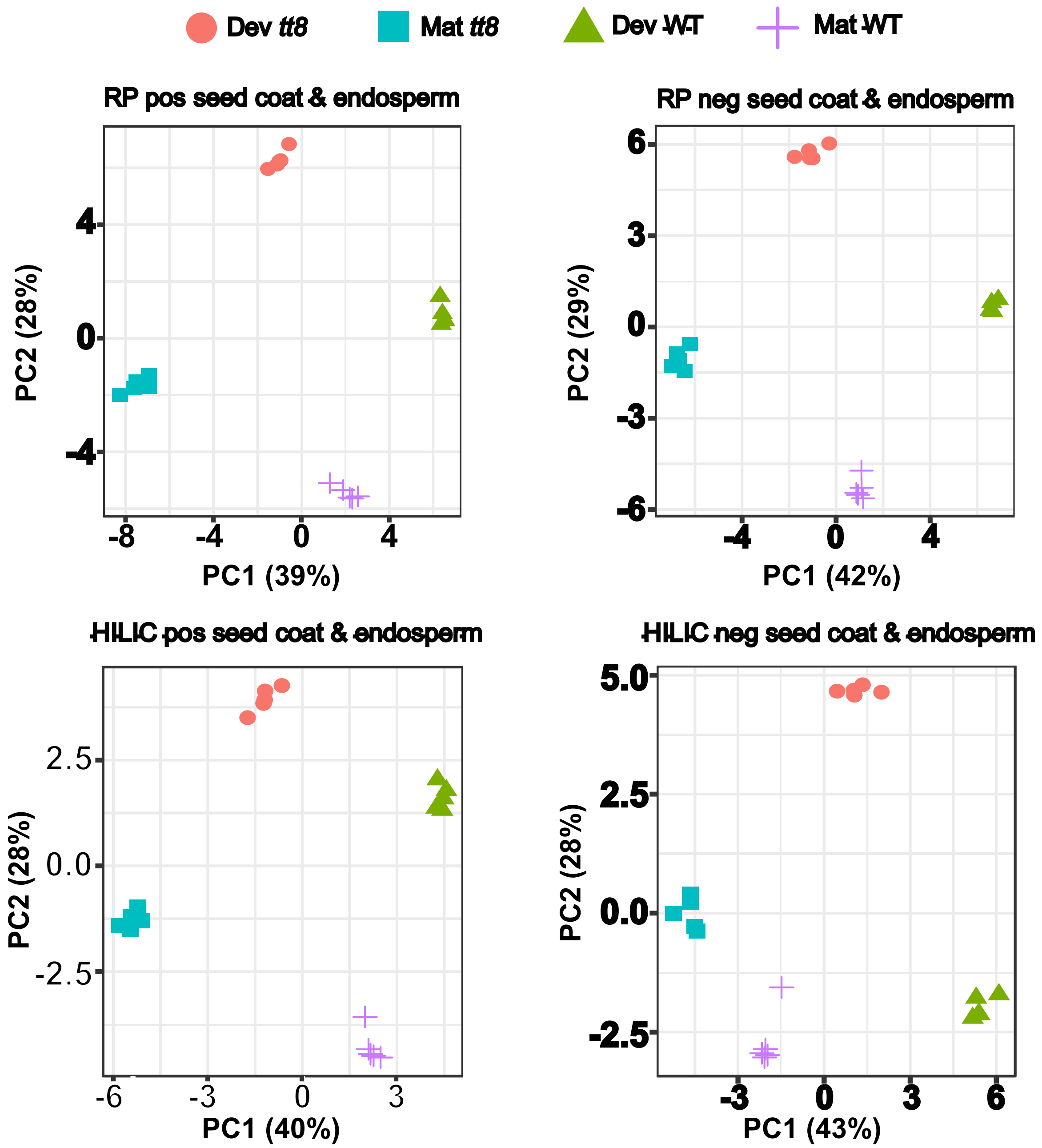

Supplementary Figure S8: Principal component analysis plots of LC-MS data of non-embryonic tissue samples analyzed under the reverse phase (RP) positive and negative modes, respectively, as well as under the Hydrophilic Interaction Liquid Chromatography (HILIC) positive and negative modes. Dev – developing (27 DAP), Mat – mature, *tt8* – *tt8-2bp*, WT – wild type, PC – principal component.

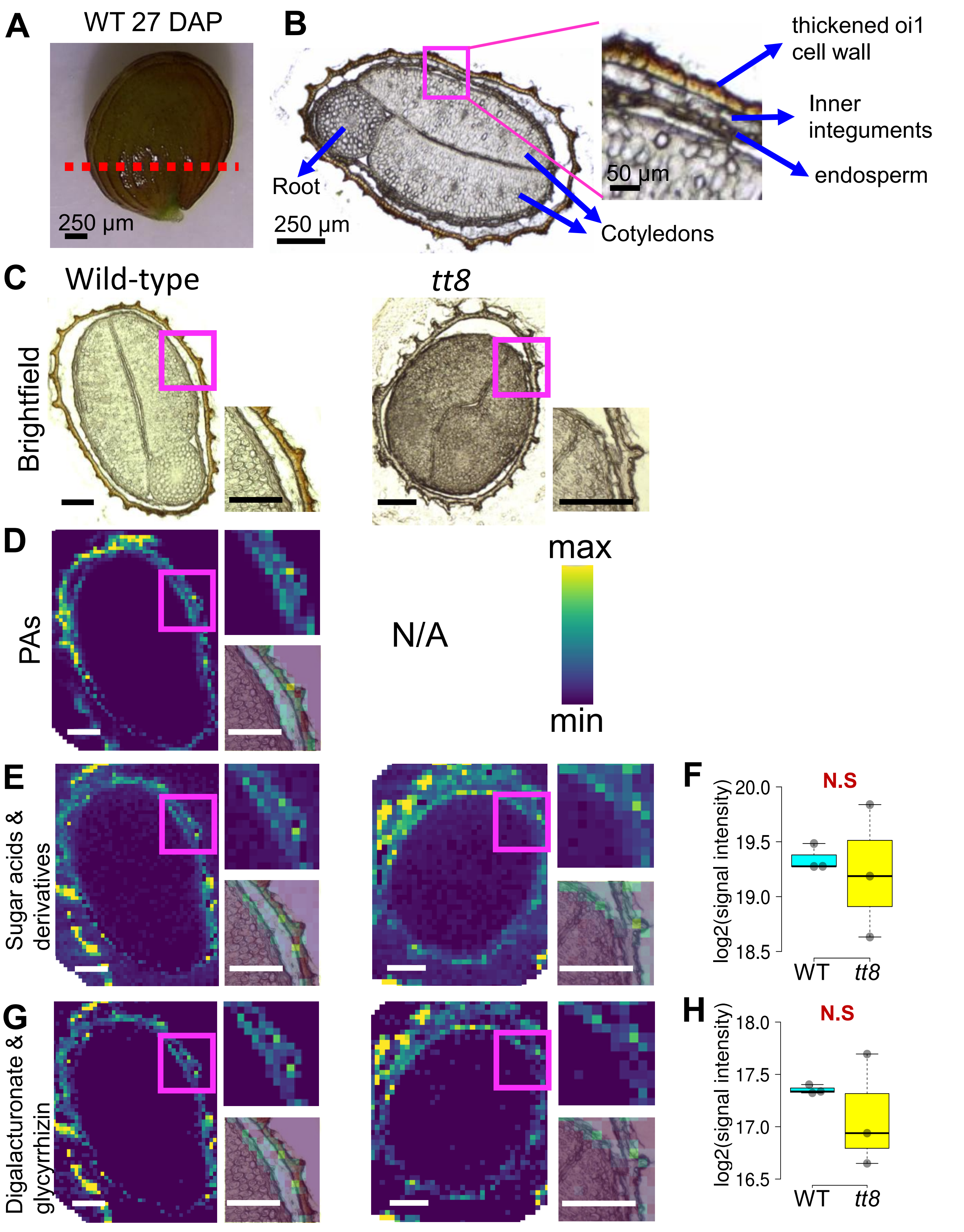
A
WT 27 DAP
250 µm
B
Root
250 µm
Cotyledons
50 µm
thickened oi1 cell wall
Inner integuments
endosperm
C
Wild-type
tt8
Brightfield
D
PAs
N/A
max
min
E
Sugar acids & derivatives
F
N.S
log2(signal intensity)
20.0
19.5
19.0
18.5
WT
tt8
G
Digalacturonate & glycyrrhizin
H
N.S
log2(signal intensity)
18.0
17.5
17.0
16.5
WT
tt8

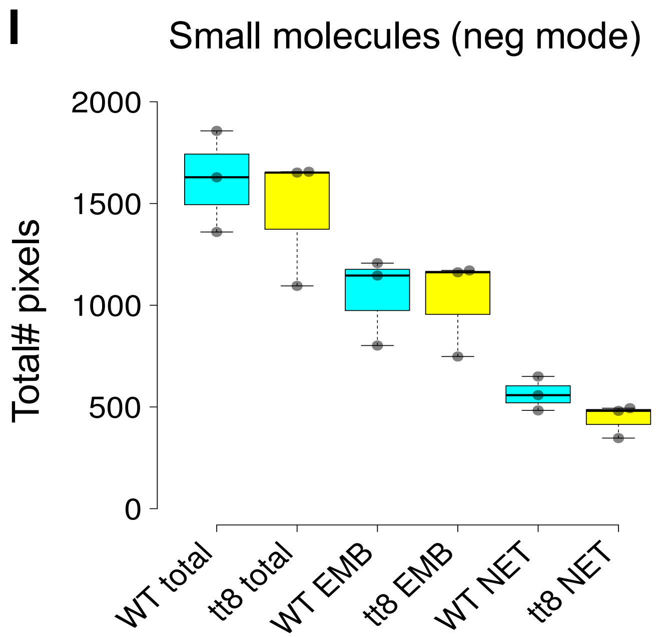

Supplementary Figure S9. Additional molecular features of interests identified in the spatial metabolomic analysis of small molecules and lipids in developing Spring32 wild-type (WT) and *tt8-2bp* (*tt8*) seeds at 27 DAP. (A) A photo of a wild-type seed at 27 DAP and the red dotted line indicate the direction of the plane where seed cryosections were acquired. (B) A brightfield micrograph of a 27 DAP WT seed cryosection (left) and the blowup image of the indicated seed area (magenta box) in which main tissues and compartments were labeled. (C) Representative images of brightfield micrographs of seed cryosections used for MALDI-MSI for the detection of small molecules under the negative ion mode. A blowup image of the indicated seed region (magenta box) is provided to show the spatial separation of embryos and non-embryonic tissues. (D, E, G) Heatmaps showing the ion signal intensities of some small molecules detected in the same seed sections show in panel C. Heatmaps of WT and *tt8* sections of the same molecular feature are displayed in the same color scale with deep blue representing the min value and yellow representing the max value. A blowup image of the indicated seed region (magenta box) is shown to the upper right of each heatmap, and an overlaid image of the blowup heatmap and the brightfield micrograph is shown to the lower right of each map. Scale bars = 200 µm. The three molecular features shown in panels D, E, and G are proanthocyanidins (PAs), sugar acids & derivatives, and digalacturonate & glycyrrhizin. PA B2, PA B4, PA B5, and epicatechin-(4beta->8)-ent-epicatechin, four different PAs, were identified for $C_{30}H_{26}O_{12}$ with m/z of 577.1351. There are 20 different sugar acids and sugar acid derivatives such as D-galacturonic acid, L-iduronic acid, *etc*. identified for $C_6H_{10}O_7$ with m/z of 193.0354. Digalacturonate and glycyrrhizin are identified for $C_{12}H_{18}O_{13}$ with m/z of 369.0675. (F, H) Boxplots of log2-transformed average ion signal intensities of molecular features detected in three wild-type and *tt8* seed sections, respectively. Two-tailed student's t-tests were performed by comparing the log2-transformed average ion signal intensities between wild-type and *tt8* seed sections. The gray dots represent values of different seed sections. N.S. means "not significant" with the significance threshold of $p<0.05$. (I) A boxplot showing the total number of pixels in the whole seed (total), embryo (EMB), and non-embryonic tissues (NET) of three wild-type and *tt8-2bp* seed sections, respectively, used for calculating average ion intensities of different molecular features. No significant difference was detected ($p<0.05$) when comparing the total number of pixels in the same seed compartment between wild-type and *tt8* seed sections using two-tailed student's t-tests.

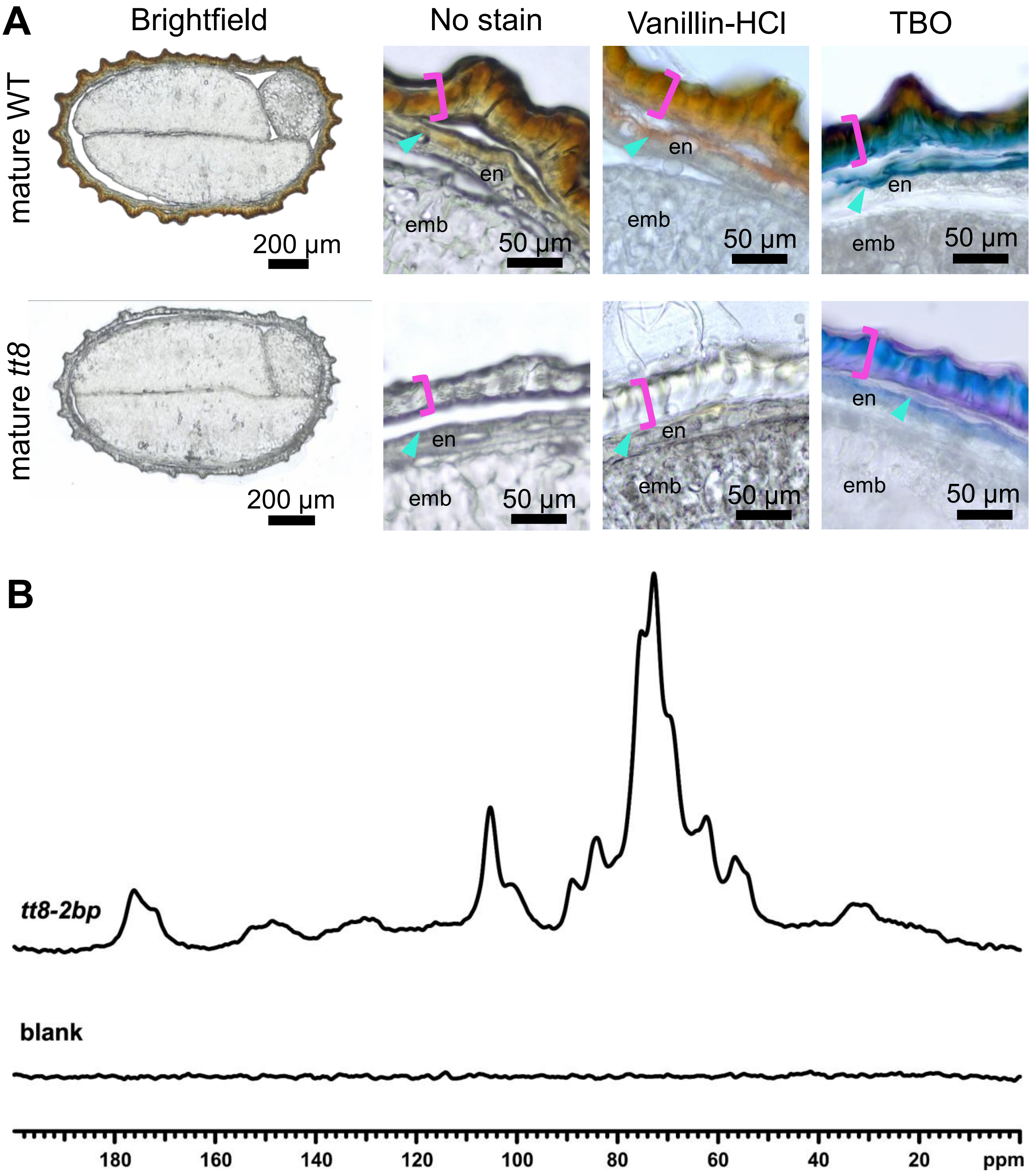
A
Brightfield
No stain
Vanillin-HCl
TBO
mature WT
mature tt8
en
emb
200 µm
50 µm
B
tt8-2bp
blank
180
160
140
120
100
80
60
40
20
ppm

Supplementary Figure S10. Cryosections of mature wild-type (WT) and *tt8-2bp* (*tt8*) seeds showing the outmost seed coat (magenta brackets) used for the ssNMR analysis and the ^{13}C CP/MAS spectra of *tt8* and a rotor full of potassium bromide served as a blank. (A) Brightfield images of unstained and stained cryosections of mature WT and *tt8* seeds. Vanillin hydrochloric acid staining (Vanillin-HCl) was used to detect proanthocyanidins which turn red when reacting with vanillin-HCl solution. Toluidine Blue O (TBO) staining was used to distinguish between primary cell wall which turns a pinkish-purple and secondary cell wall and aromatic compounds including proanthocyanidins which turn a blueish-green after staining. The magenta brackets mark the outmost seed coats derived mainly from the thickened oi1 cell wall which was manually dissected and used for ss NMR and the turquoise arrowheads point to the ii1 layer. en – endosperm, emb – embryo. Note that the empty space between the ii1 and the outmost seed coat of wild-type and *tt8* seeds resulted from the dead cells of ii2 and ii3 upon seed maturity, which allowed for easy dissection and isolation of the outmost seed coats. (B) Both spectra were acquired under identical conditions and are plotted on the same scale. Recycle delay was 2s and total number of transients was 65536 for each.
